# Upregulated endonuclease Regnase-1 suppresses osteoarthritis by forming a negative feedback loop of catabolic signaling in chondrocytes

**DOI:** 10.1186/s13075-021-02485-z

**Published:** 2021-04-14

**Authors:** Jeong-In Yang, Jang-Soo Chun

**Affiliations:** National Creative Research Initiatives Center for Osteoarthritis Pathogenesis and School of Life Sciences, Gwangju Institute of Science and Technology, Gwangju, 61005 Republic of Korea

**Keywords:** Regnase-1, Chondrocytes, Cartilage, Osteoarthritis, Matrix-degrading enzymes

## Abstract

**Background:**

Ribonucleases (RNases) play central roles in the post-transcriptional regulation of mRNA stability. Our preliminary results revealed that the endonuclease Regnase-1 is specifically upregulated in osteoarthritic chondrocytes. We herein explored the possible functions and regulatory mechanisms of Regnase-1 in a mouse model of osteoarthritis (OA).

**Methods:**

The expression and target genes of Regnase-1 were identified by microarray analysis in primary-culture mouse articular chondrocytes. Experimental OA in mice was induced by destabilization of the medial meniscus (DMM). The function of Regnase-1 in DMM-induced post-traumatic OA mice was examined by adenovirus-mediated overexpression or knockdown in knee joint tissues, and also by using Regnase-1 heterozygous knockout mice (*Zc3h12a*^*+/−*^).

**Results:**

Among the RNases, Regnase-1 was exclusively upregulated in chondrocytes stimulated with OA-associated catabolic factors. Adenovirus-mediated overexpression or knockdown of Regnase-1 alone in joint tissues did not cause OA-like changes. However, overexpression of Regnase-1 in joint tissues significantly ameliorated DMM-induced post-traumatic OA cartilage destruction, whereas knockdown or genetic ablation of Regnase-1 exacerbated DMM-induced cartilage destruction. Mechanistic studies suggested that Regnase-1 suppresses cartilage destruction by modulating the expression of matrix-degrading enzymes in chondrocytes.

**Conclusion:**

Our results collectively suggest that upregulated Regnase-1 in OA chondrocytes may function as a chondro-protective effector molecule during OA pathogenesis by forming a negative feedback loop of catabolic signals, such as matrix-degrading enzyme expression, in OA chondrocytes.

**Supplementary Information:**

The online version contains supplementary material available at 10.1186/s13075-021-02485-z.

## Background

Osteoarthritis (OA) is a whole-joint disease characterized by cartilage destruction, synovial inflammation, osteophyte formation, subchondral bone remodeling, etc. [1]. Among these manifestations of OA, progressive articular cartilage degradation is a hallmark of OA. This degradation is primarily regulated by chondrocytes through the production of matrix-degrading enzymes and/or the downregulation of cartilage extracellular matrix (ECM) molecules [2]. Among the matrix-degrading enzymes, matrix metalloproteinase 3 (MMP3), MMP13, and ADAMTS5 are known to play important roles in OA cartilage destruction [3–5]. In chondrocytes, the expression levels of these molecules are regulated by extracellular catabolic regulators, such as the pro-inflammatory cytokine, and interleukin (IL)-1β [6]. We previously identified cellular catabolic mediators in chondrocytes, including the transcription factor, hypoxia-inducible factor (HIF)-2α [7], the zinc importer, ZIP8 [8], and the cholesterol hydroxylase, CH25H [9]. These cellular mediators exert catabolic functions by upregulating matrix-degrading enzymes and/or downregulating ECM molecules in chondrocytes.

The expression of OA-related catabolic and anabolic factors in chondrocytes can be regulated at multiple checkpoints, including transcriptional and post-transcriptional levels. The ribonucleases (RNases) comprise a broad class of RNA-degrading enzymes that play central roles in the post-transcriptional regulation of mRNA stability [10]. However, the roles of specific ribonucleases and their targets in OA pathogenesis have not yet been clearly elucidated. Here, we initially used a microarray analysis to screen for RNases whose expression levels were modulated in chondrocytes upon stimulation with OA-associated catabolic factors, such as IL-1β treatment, ZIP8 overexpression, or HIF-2α overexpression [6–8]. Among the examined RNases, our initial screening analysis revealed that Regnase-1 (a zinc finger CCCH-type containing 12A encoded by *Zc3h12a* in mouse) was exclusively upregulated in chondrocytes stimulated with OA-associated catabolic factors. Regnase-1, which is also known as MCPIP1 (monocyte chemotactic protein-induced protein 1), is an endonuclease that destabilizes various target mRNAs by recognizing a stem-loop structure within their 3′-UTRs [11, 12]. Regnase-1 is known to play diverse functions in different cell types, such as mitigating inflammation by downregulating the IL-6 and IL-12B mRNAs in monocytes [11]; restricting T cell activation by targeting the c-Rel, Ox40, and IL-2 mRNAs in T cells [13, 14]; and promoting cancer cell apoptosis by regulating the Bcl2L1, Bcl2A1, RelB, and Bcl3 mRNAs [15].

One of the known Regnase-1 targets in chondrocytes is IL-6 [16, 17], which plays an important role in OA pathogenesis [18, 19]. However, the role of Regnase-1 in OA pathogenesis in vivo has not been investigated to date. Thus, we herein explored the possible functions and regulatory mechanisms of Regnase-1 in mouse models of post-traumatic OA. Our gain-of-function (adenovirus-mediated overexpression in joint tissues) and loss-of-function (*Zc3h12a*^*+/−*^) approaches clearly indicated that upregulated Regnase-1 in OA chondrocytes exerts chondro-protective functions at least partly by modulating the expression levels of matrix-degrading enzymes (MMP3, MMP13, and ADAMTS5) in articular chondrocytes.

## Methods

### Mice and experimental OA

C57BL/6 J mice were used for the experimental OA studies. C57BL/6 J-background Regnase-1-KO mice (68-bp deletion in exon 2) were generated by ToolGen, Inc. (Seoul, Korea). Because homozygous KO mice (*Zc3h12a*^*−/−*^) die shortly after weaning [20], we used heterozygous *Zc3h12a*^*+/−*^ mice for our experimental OA studies. All experiments were approved by the Animal Care and Use Committee of Gwangju Institute of Science and Technology. Post-traumatic OA was induced in 12-week-old male mice by destabilization of the medial meniscus (DMM), and the mice were sacrificed at the indicated weeks after the surgery [21]. Experimental OA was also induced by IA injection (once weekly for 3 weeks) of adenovirus [1 × 10^9^ plaque-forming units (PFUs) in a total volume of 10 μl] expressing Regnase-1 (Ad-Regnase-1), SAA3 (Ad-SAA3), ZIP8 (Ad-ZIP8), or HIF-2α (Ad-HIF-2α). Mice were sacrificed 3 weeks after the first injection of adenovirus [7–9]. When IA injection was performed after DMM surgery, the injections were initiated at 10 days after surgery and performed weekly thereafter for a total of three injections. The mice were sacrificed at the indicated weeks after the surgery.

### Histological analysis

Mouse knee joint samples were fixed with 4% paraformaldehyde, decalcified in 0.5 M EDTA, embedded in paraffin, and sectioned frontally at 5-μm thickness. Sections were deparaffinized in xylene, hydrated with graded ethanol, and stained with Safranin-O [8, 9]. Scores of OARSI grade and synovitis were calculated as the average values obtained from three different sections selected at ~ 100-μm intervals for each knee joint; each section was scored by four observers under blinded conditions. OARSI grade (0–6) was expressed as the maximum score observed among the medial femoral condyle, medial tibial plateau, lateral femoral condyle, and lateral tibial plateau [22]. Synovitis was determined by Safranin-O and hematoxylin staining, and synovial inflammation (grade 0–3) was scored [23]. Osteophytes were identified by Safranin-O staining, and osteophyte size was measured with an Aperio ImageScope (Leica Biosystems) [4]. We measured the thickness of the subchondral bone plate (SBP) using an Aperio ImageScope to assess subchondral bone sclerosis [24]. The skeletons of E18.5 whole-mouse embryos were stained with Alcian blue and Alizarin red [8, 9]. The following antibodies were used for immunostaining of cartilage and synovial sections: rabbit anti-Regnase-1 (Novus Biologicals), rabbit anti-MMP3 (Abcam), and rat anti-SAA3 (Abcam).

### Primary culture of mouse articular chondrocytes and fibroblast-like synoviocytes (FLS)

Mouse articular chondrocytes were isolated from the femoral condyles and tibial plateaus of 5-day-old WT or *Zc3h12a*^*−/−*^ mice by 0.2% collagenase digestion [25]. The cells were maintained as a monolayer in Dulbecco’s modified Eagle’s medium (DMEM; Gibco) supplemented with 10% fetal bovine serum and antibiotics (penicillin G and streptomycin). On culture day 2, cells were infected with the indicated MOI (multiplicity of infection) of empty adenovirus (Ad-C), Ad-Regnase-1, Ad-SAA3, Ad-HIF-2α, Ad-ZIP8, Ad-shScramble, or Ad-shRegnase-1, for 2 h and either maintained for an additional 36 h prior to further biochemical analysis or treated with IL-1β as indicated in each experiment. Mouse FLS were isolated and cultured as described previously [8, 9]. FLS of passages 4–8 were used for analyses. Pure FLS (> 90% CD90^+^/< 1% CD14^+^) were identified by flow cytometry using antibodies against CD90 and CD14 (Abcam). The cells were maintained in DMEM supplemented with 10% fetal bovine serum and antibiotics. FLS were treated with reagents as indicated in each experiment or infected with the indicated adenovirus.

### Apoptosis assay

Primary-culture chondrocytes were infected with indicated MOI of Ad-C, Ad-Regnase-1, or Ad-shRegnase-1 for 36 h. The cells were exposed to the nitric oxide (NO) donor, sodium nitroprusside (SNP), to induce apoptosis as a positive control [26]. Apoptosis markers (e.g., Fas, Bad, and Bcl3) were detected by RT-PCR analysis. To examine apoptosis of chondrocytes in the cartilage tissue, sham- or DMM-operated WT mice were IA injected with Ad-C, Ad-shScramble, Ad-Regnase-1, or Ad-shRegnase-1 to modulate Regnase-1 expression. Apoptotic chondrocytes in the cartilage tissue were detected by a TUNEL (terminal deoxynucleotidyl transferase dUTP nick-end labeling) assay kit from Roche Diagnostics [27].

### Western blotting

Total cell lysates were prepared in lysis buffer [150 mM NaCl, 1% NP-40, 50 mM Tris, 0.2% sodium dodecyl sulfate (SDS), 5 mM NaF] containing a cocktail of protease inhibitors and phosphatase inhibitors (Roche). The following antibodies were used for Western blotting to detect cellular proteins: rabbit anti-Regnase-1 (Novus Biologicals), rabbit anti-SOX9 (Abcam), mouse anti-HIF-2α (Santa Cruz Biotech), and goat anti-Lamin B (Santa Cruz Biotech). For detection of secreted proteins (MMP3 and MMP13), 900 μl of serum-free conditioned medium was subjected to trichloroacetic acid precipitation, and the proteins were fractionated by SDS-PAGE, transferred to a nitrocellulose membrane, and detected using rabbit anti-MMP3 (Abcam) and rabbit anti-MMP13 (Aviva Systems Biology) antibodies.

### Enzyme-linked immunosorbent assay (ELISA)

The protein levels of all SAA isoforms secreted from primary-culture chondrocytes were detected by ELISA. Briefly, 900 μl of serum-free-conditioned medium were collected and stored at − 80 °C until analysis. The SAA levels were analyzed using a mouse quantikine ELISA kit (R&D systems) according to the manufacturer’s protocol.

### Microarray analysis

Our microarray data from chondrocytes stimulated with IL-1β treatment or those overexpressing HIF-2α (Ad-HIF-2α infection) or ZIP8 (Ad-ZIP8 infection) were previously deposited to the Gene Expression Omnibus under accession codes GSE104794 (HIF-2α), GSE104795 (ZIP8), and GSE104793 (IL-1β). We also performed microarray analysis in chondrocytes infected with 800 MOI of Ad-Regnase-1 or Ad-shRegnase-1 for 36 h. Briefly, the total RNA was extracted from mouse articular chondrocytes using a Purelink RNA mini kit (Ambion) and analyzed using Affymetrix Gene Chip arrays (Affymetrix GeneChip Mouse Gene 2.0 ST Array) in accordance with the Affymetrix protocol (Macrogen Inc.). The probe signals in the raw data were normalized with respect to the RMA (Robust Multi-array Average) for each separate data set (infection of Ad-C, Ad-Regnase-1, Ad-shC, or Ad-shRegnase-1). These microarray data were deposited to the Gene Expression Omnibus under accession codes GSE153179 (for Ad-Regnase-1 and Ad-shRegnase-1).

### Reverse transcription-polymerase chain reaction (RT-PCR) and quantitative RT-PCR (qRT-PCR) analysis

Total RNA was extracted from primary-culture chondrocytes using the TRI reagent (Molecular Research Center, Inc.). The total RNA was reverse transcribed, and the resulting cDNA was PCR amplified using the PCR primers and experimental conditions summarized in Supplementary Table 1. qRT-PCR was performed using an iCycler thermal cycler (Bio-Rad) and SYBR premixExTaq. Transcript levels were normalized with respect to those of β-actin and expressed as fold changes relative to the control.

### Luciferase assay

The SAA3–3′-UTR reporter plasmid, which contained the 5′-AATAAATACTTGTGAAATGCA-3′ sequence of 3′-UTR of SAA3, was purchased from GeneCopoeia. Primary-culture mouse chondrocytes were pretreated with hyaluronidase type I-S (Sigma) for 3 h in serum-free DMEM and transfected by incubation for 6 h with SAA3-3′-UTR reporter vector (0.05 μg) and Lipofectamine 2000, as described by the manufacturer. The cells were co-transfected with 0.05 μg of empty vector (Origene), WT-Regnase-1 expression vector (Origene), or D141N Regnase-1 (which lacks RNase activity) [13]. The cells were harvested at 24 h after treatment, and firefly luciferase and Renilla luciferase activities were measured using a Dual-Luciferase Assay System (Promega).

### Statistical analysis

For statistical comparison of experimental groups, the data were analyzed by the Shapiro-Wilk test for normality and Levene’s test for homogeneity of variance. Two groups of non-parametric data based on the ordinal grading systems (OARSI grade, synovitis, osteophyte maturity) were compared using the Mann–Whitney *U* test, whereas the Kruskal-Wallis test was used to compare multi-groups of non-parametric data. We also used the Mann–Whitney *U* test for the direct comparison of pairs of groups among the multi-groups of non-parametric data. Parametric data collected from two independent experimental groups were compared by two-tailed *t* test. For comparisons of three or more groups of parametric data, one-way analysis of variance (ANOVA) with Bonferroni’s post hoc test was used. Each *n* number indicates the number of biologically independent samples or mice per group. Significance was accepted at the 0.05 level of probability (*p* < 0.05). Each bar represents the s.e.m. for parametric data and the calculated 95% confidence interval (CI) for nonparametric data.

## Results

### OA-associated catabolic factors upregulate Regnase-1 in mouse articular chondrocytes

**Fig. 1:**
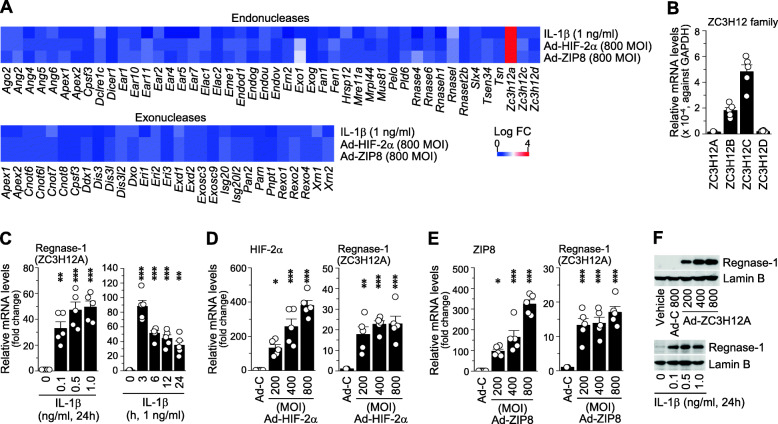
Regnase-1 is upregulated in chondrocytes by OA-associated catabolic factors. **a** Heat map of RNase mRNA expression levels obtained by microarray analysis of primary-culture mouse articular chondrocytes treated with IL-1β, Ad-HIF-2α, or Ad-ZIP8 (*n* = 4). **b** Relative mRNA levels of ZC3H12 family members in unstimulated chondrocytes (*n* = 5). **c**–**e** Relative mRNA levels of Regnase-1 and the indicated molecules in chondrocytes stimulated with IL-1β (**c**), infected with Ad-HIF-2α (**d**), or infected with Ad-ZIP8 (**e**) (*n* = 5). **f** Representative Western blot images of Regnase-1 in chondrocytes treated with Ad-Regnase-1 or IL-1β (*n* = 4). Lamin B was used as a loading control. Values in **b**–**e** are means ± s.e.m. and one-way ANOVA with Bonferroni’s post hoc test (*p < 0.05, **p < 0.005, ***p < 0.0005)

To identify RNases that may be associated with OA pathogenesis, we analyzed the expression levels of RNases (both endonucleases and exonucleases) in primary-culture articular chondrocytes stimulated with OA-associated catabolic factors, including a pro-inflammatory cytokine (IL-1β) [6] and adenovirus-mediated overexpression of HIF-2α (Ad-HIF-2α) [7] or ZIP8 (Ad-ZIP8) [8]. Our microarray analysis revealed that Regnase-1 (encoded by *Zc3h12a*) was exclusively upregulated in chondrocytes stimulated with the tested catabolic regulators (Fig. 1a, Supplementary Table 2). We further characterized the expression of Regnase-1 in primary-culture chondrocytes. Unstimulated chondrocytes exhibited various mRNA levels of the different ZC3H12 family members (Fig. 1b); our RT-PCR (Supplementary Fig. 1A and B) and quantitative RT-PCR (qRT-PCR) (Fig. 1c–e) analyses revealed that Regnase-1 was upregulated by IL-1β treatment (Fig. 1c) or infection of Ad-HIF-2α (Fig. 1d) or Ad-ZIP8 (Fig. 1e). Regnase-1 protein levels were also increased by IL-1β treatment or Ad-Regnase-1 infection in primary-culture chondrocytes (Fig. 1f). The mRNA levels of other ZC3H12 family members were not modulated by Regnase-1 overexpression (Supplementary Fig. 1C). Although articular chondrocytes play a critical role in OA pathogenesis, other joint cell types, such as FLS in synovium, also play roles in the onset and progression of OA [1, 2]. We, therefore, examined the effects of OA-associated catabolic factors on the expression of ZC3H12 family members in FLS. All of the examined catabolic factors caused specific upregulation of Reganse-1 in FLS, although the degree of upregulation was much smaller than that observed in chondrocytes (Supplementary Fig. 1D and E). Additionally, overexpression or downregulation of Regnase-1 in FLS by infection Ad-Regnase-1 and Ad-shRegnase-1, respectively, did not alter the expression levels of the tested matrix-degrading enzymes (Supplementary Fig. 1F). We, therefore, focused on exploring the function of Regnase-1 in chondrocytes during OA pathogenesis.

### Overexpression of Regnase-1 in joint tissues ameliorates post-traumatic OA cartilage destruction in mice

**Fig. 2:**
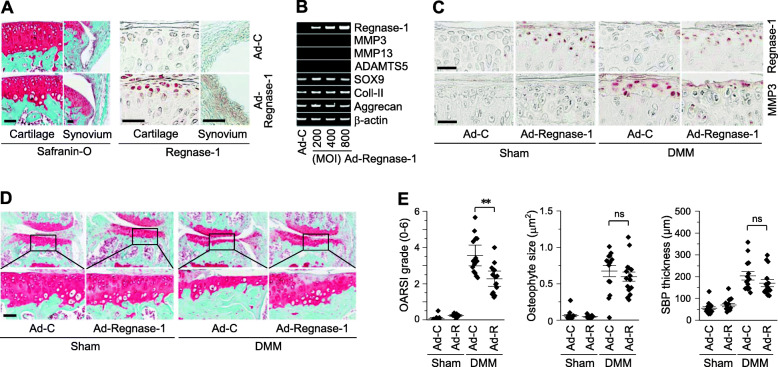
Overexpression of Regnase-1 in joint tissue ameliorates post-traumatic OA cartilage destruction in mice. **a** Representative images of Safranin-O staining (left) and Regnase-1 immunostaining (right) of WT mice IA-injected with empty virus (Ad-C; *n* = 5 mice) or Ad-Regnase-1 (*n* = 8 mice). **b** Representative RT-PCR images of the indicated molecules in chondrocytes treated with the indicated MOI of Ad-C or Ad-Regnase-1 (*n* = 5). **c**–**e** Sham- or DMM-operated WT mice were IA-injected with Ad-Control (Ad-C) or Ad-Regnase-1 (Ad-R). Representative immunostaining images of Regnase-1 and MMP3 in cartilage sections (**c**), Safranin-O staining images (**d**), and scoring of OARSI grade, osteophyte size, and subchondral bone plate (SBP) thickness (**e**) at 8 weeks after operations (*n* = 15 mice per group). Vales in **e** are means ±95% CI with Mann–Whitney *U* test for OARSI grade and means ± s.e.m. with two-tailed *t* test for osteophyte size and SBP thickness. **p < 0.005. ns, not significant. Scale bar: 50 μm

Because the above-described results suggest that upregulated Regnase-1 in OA-like chondrocytes may play a role in OA pathogenesis, we examined the functions of Regnase-1 in OA development by overexpressing it in mouse knee joint tissues via intra-articular (IA) injection of Ad-Regnase-1. We previously reported that an adenoviral system can be used to effectively deliver transgenes into joint tissues [7–9]. Consistent with this, immunohistochemical staining of joint sections confirmed the overexpression of Regnase-1 in cartilage and synovial tissues (Fig. 2a). However, this overexpression of Regnase-1 did not cause any OA-like change in the cartilage or synovium of the joint tissues (Fig. 2a). Consistently, Regnase-1 overexpression in primary-culture chondrocytes did not affect the mRNA levels of matrix-degrading enzymes (MMP3, MMP13, and ADAMTS5) or cartilage ECM molecules (type II collagen or aggrecan) (Fig. 2b).

We further examined the effects of Regnase-1 overexpression in knee joint tissues under DMM-induced post-traumatic OA. In contrast to the effects of Regnase-1 overexpression alone, adenoviral overexpression of Regnase-1 in joint tissues in DMM-operated mice (Fig. 2c) significantly suppressed post-traumatic OA cartilage destruction (Fig. 2d, e). Other examined manifestations of OA, such as osteophyte formation and thickness of the subchondral bone plate (SBP), also tended to be reduced by Regnase-1 overexpression, but the differences did not reach the level of statistical significance (Fig. 2d, e). Consistent with this reduced OA cartilage destruction, the increased expression of MMP3 protein in cartilage of DMM-operated mice was reduced by Regnase-1 overexpression via IA injection of Ad-Regnase-1 (Fig. 2c). These findings suggest that overexpression of Regnase-1 alone does not cause OA pathogenesis; instead, upregulated Regnase-1 in OA chondrocytes appears to play a protective role in DMM-induced post-traumatic cartilage destruction in mice.

### Knockdown of Regnase-1 exacerbates post-traumatic OA cartilage destruction

**Fig. 3:**
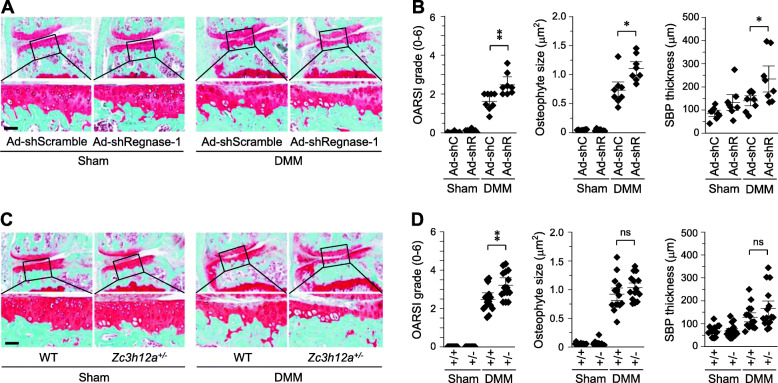
Knockdown or genetic ablation of Regnase-1 exacerbates OA pathogenesis in mice. **a**, **b** Sham- or DMM-operated WT mice were IA-injected with Ad-shScramble (Ad-shC) or Ad-shRegnase-1 (Ad-shR) and sacrificed at 6 weeks after operation (*n* = 8 mice per group). Safranin-O staining images (**a**) and scoring of OARSI grade, osteophyte size, and SBP thickness (**b**). **c**, **d** WT (*Zc3h12a*^*+/+*^) and *Zc3h12a*^*+/−*^ mice were subjected to sham operation or DMM surgery and sacrificed at 6 weeks after operation (*n* = 15 mice per group). Representative Safranin-O staining images (**c**) and scoring of OARSI grade, osteophyte size, and SBP thickness (**d**). Values in **b** and **d** are means ±95% CI with Mann–Whitney *U* test for OARSI grade and means ± s.e.m. with two-tailed *t* test for osteophyte size and SBP thickness. *p < 0.05 and **p < 0.005. ns, not significant. Scale bar: 50 μm

To further elucidate the functions of Regnase-1 in OA pathogenesis, we downregulated Regnase-1 in whole-joint tissues via IA injection of an adenovirus expressing shRNA against Regnase-1 (Ad-shRegnase-1). IA injection of Ad-shRegnase-1 alone did not cause cartilage damage (Supplementary Fig. 2A). Consistently, Regnase-1 knockdown in primary-culture chondrocytes did not affect the expression levels of matrix-degrading enzymes, cartilage ECM molecules, or other members of ZC3H12 family (Supplementary Fig. 2B and C). However, knockdown of Regnase-1 in joint tissues via IA injection of Ad-shRegnase-1 in DMM-operated mice (Supplementary Fig. 2D) significantly enhanced DMM-induced OA manifestations, such as cartilage destruction, osteophyte formation, and thickening of the SBP plate (Fig. 3a, b). Furthermore, the increased MMP3 protein level found in cartilage of DMM-operated mice was further increased by Regnase-1 knockdown via IA injection of Ad-shRegnase-1 (Supplementary Fig. 2D). Our results are consistent with the idea that upregulated Regnase-1 in OA chondrocytes has chondro-protective effects in DMM-induced OA pathogenesis.

We further validated the functions of Regnase-1 in OA pathogenesis by using a Regnase-1 KO mouse generated by deleting 68 bp from exon 2 of the *Zc3h12a* gene (Supplementary Fig. 3A and B). Because homozygous KO mice (*Zc3h12a*^*−/−*^) exhibit pre-mature death within 12 weeks of birth [20], we used heterozygous *Zc3h12a*^*+/−*^ mice for our experimental OA studies. *Zc3h12a*^*+/−*^ mice exhibited markedly decreased mRNA levels of Regnase-1, but no alteration of the mRNA levels for other ZC3H12 family members, in chondrocytes and had normal skeletal development in E18.5 embryos (Supplementary Fig. 3C). Compared with WT littermates, *Zc3h12a*^*+/−*^ mice exhibited significant enhancement of DMM-induced cartilage erosion but no significant change in osteophyte formation or SBP thickness (Fig. 3c, d). Consistently, the increased MMP3 protein level in the cartilage of DMM-operated WT mice was further increased in *Zc3h12a*^*+/−*^ mice (Supplementary Fig. 3D). These results additionally indicate that upregulated Regnase-1 exerts protective functions in DMM-induced post-traumatic OA cartilage destruction in mice.

### Regnase-1 modulates the expression levels of matrix-degrading enzymes in chondrocytes

**Fig. 4:**
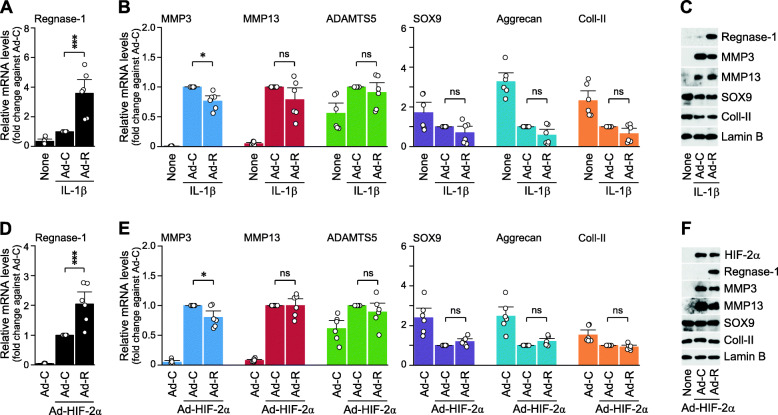
Effects of Regnase-1 overexpression on the expression of matrix-degrading enzymes and ECM molecules. **a**–**c** Primary-culture chondrocytes were infected with 800 MOI of Ad-C or Ad-Regnase-1 (Ad-R) for 12 h, and exposed to IL-1β (0.01 ng/ml) for an additional 24 h. mRNA levels of the indicated molecules were quantified by qRT-PCR analysis (**a**, **b**, *n* = 6). **d**–**f** Primary-culture chondrocytes were pre-infected with 400 MOI of Ad-C or Ad-Regnase-1 (Ad-R) for 12 h and additionally infected with 400 MOI of Ad-HIF-2α for 24 h. Untreated chondrocytes were infected with 800 MOI of Ad-C as a control. The indicated molecules were quantified by qRT-PCR analysis (**d**, **e**, *n* = 6). Representative Western blot images of the indicated molecules are presented in **c** (*n* = 6). Means ± s.e.m. with one-way ANOVA with Bonferroni’s post hoc test. *p < 0.05 and ***p < 0.0005. ns, not significant

To elucidate possible mechanisms underlying the protective effects of Regnase-1, we examined whether Regnase-1 modulates the expression levels of matrix-degrading enzymes and cartilage ECM molecules in chondrocytes. For this purpose, Regnase-1 was overexpressed in primary-culture chondrocytes via Ad-Regnase-1 infection, and the chondrocytes were stimulated with IL-1β (Fig. 4a). Among the examined molecules, the mRNA and protein levels of MMP3 were slightly but significantly decreased by the overexpression of Regnase-1 in IL-1β-treated chondrocytes (Fig. 4b and c). Similarly, the HIF-2α-induced increases in the mRNA and protein levels of MMP3 were slightly but significantly decreased by Regnase-1 overexpression, whereas the mRNA levels of the other examined catabolic and anabolic factors were not modulated (Fig. 4d–f).

**Fig. 5:**
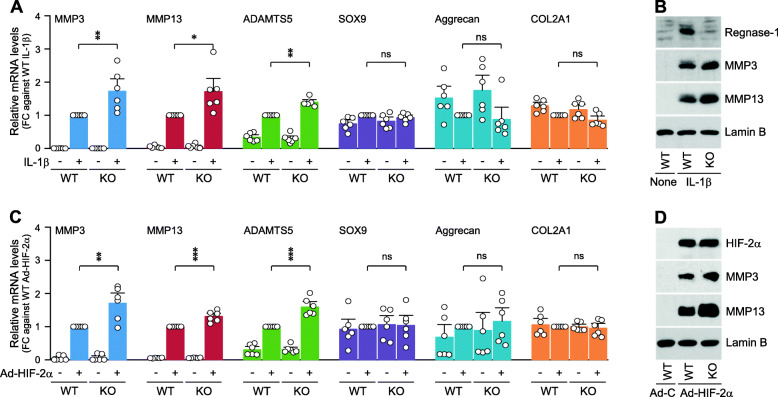
*Zc3h12a*^*−/−*^ chondrocytes exhibit enhanced expression of matrix-degrading enzymes when treated with IL-1β or Ad-HIF-2α. **a**, **b** WT and KO (*Zc3h12a*^*−/−*^) chondrocytes were treated with 0.01 ng/ml of IL-1β for 36 h mRNA levels (**a**) and protein levels (**b**) of the indicated molecules were determined by qRT-PCR analysis and Western blotting, respectively (*n* = 6). **c**, **d** WT and KO (*Zc3h12a*^*−/−*^) chondrocytes were infected with 800 MOI of Ad-HIF-2α for 36 h. mRNA levels (**c**) and protein levels (**d**) of the indicated molecules were determined by qRT-PCR analysis and Western blotting, respectively (*n* = 6). Values are means ± s.e.m. with one-way ANOVA with Bonferroni’s post hoc test. *p < 0.05, **p < 0.005, ***p < 0.0005. ns, not significant

While Regnase-1 overexpression caused a relatively small modulation of MMP3 expression, *Zc3h12a*^*−/−*^ chondrocytes exhibited a more dramatic modulation of the expression levels of matrix-degrading enzymes: *Zc3h12a*^*−/−*^ chondrocytes exhibited significantly increased expression of MMP3, MMP13, and ADAMTS5 at the mRNA and protein levels under IL-1β treatment, compared to the levels seen in IL-1β-treated WT chondrocytes (Fig. 5a, b). The upregulations of MMP3, MMP13, and ADAMTS5 in HIF-2α-overexpressing chondrocytes were also significantly enhanced in *Zc3h12a*^*−/−*^ chondrocytes (Fig. 5c, d). The enhanced expression levels of matrix-degrading enzymes in *Zc3h12a*^*−/−*^ chondrocytes were well consistent with the enhanced OA cartilage destruction seen in DMM-operated *Zc3h12a*^*−/−*^ mice.

Although overexpression or knockdown of Regnase-1 alone did not modulate the expression levels of matrix-degrading enzymes in chondrocytes (Fig. 2b, Supplementary Fig. 2B), the upregulation of these enzymes by OA-associated catabolic factors, such as IL-1β and HIF-2α, was significantly inhibited by Regnase-1 overexpression (Fig. 4) and potentiated by Regnase-1 knockout in chondrocytes (Fig. 5). Therefore, it is likely that upregulated Regnase-1 in OA chondrocytes may suppress cartilage destruction by forming a negative feedback loop of catabolic signals, such as the expression of matrix-degrading enzymes, in chondrocytes. This idea was further supported by our observation that the MMP3 protein level in the cartilage of DMM-operated mice was reduced by Regnase-1 overexpression (Fig. 2c), whereas the DMM-induced upregulation of MMP3 protein in the cartilage tissue was further increased by Regnase-1 knockdown (Supplementary Fig. 2D) and in *Zc3h12a*^*+/−*^ mice compared with WT littermates (Supplementary Fig. 3D).

### Regnase-1 does not modulate chondrocyte apoptosis

A previous report that Regnase-1 selectively destabilizes transcripts associated with antiapoptotic genes in breast cancer cells [15] led us to examine whether Regnase-1 modulates chondrocyte apoptosis during OA pathogenesis. In contrast to cancer cells, either upregulation or downregulation of Regnase-1 in primary-culture chondrocytes via infection of Ad-Regnase-1 and Ad-shRegnase-1, respectively, did not modulate the mRNA levels of apoptosis-associated Fas, Bad, or Bcl3 (Supplementary Fig. 4A). Furthermore, IA injection of Ad-Regnase-1 or Ad-shRegnase-1 in mice did not modulate the DMM-induced apoptosis of articular chondrocytes (Supplementary Fig. 4B and C). These results indicate that chondrocyte apoptosis is not associated with the capacity of Regnase-1 to modulate OA pathogenesis in mice.

### Identification and characterization of Regnase-1 targets in chondrocytes

**Fig. 6:**
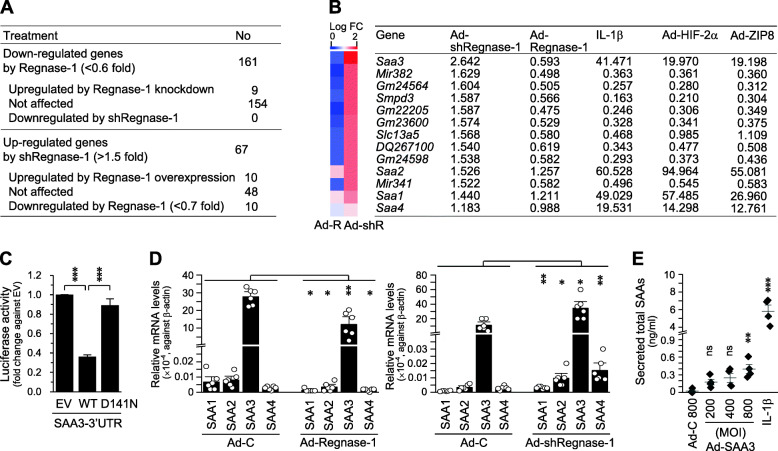
SAA3 is a target of Regnase-1 in chondrocyte. **a**, **b** Number of genes modulated by Regnase-1 (**a**) and list of the top 13 genes that were most markedly regulated by Ad-shRegnase-1 (**b**). **c** Luciferase assay (*n* = 4) in chondrocytes transfected with a reporter plasmid containing the SAA3 3′-UTR sequence. Chondrocytes were co-transfected with empty vector (EV), WT Regnase-1 (WT), or D141N Regnase-1 (D141N). **d** qRT-PCR analysis (*n* = 6) of SAA family members in chondrocytes infected with Ad-Regnase-1 or Ad-shRegnase-1 for 36 h. **e** Quantitation of secreted SAAs by ELISA in chondrocytes treated with IL-1β (0.1 ng/ml, 36 h) or infected with the indicated MOI of Ad-C or Ad-SAA3 (36 h) (*n* = 4). Means ± s.e.m. and one-way ANOVA with Bonferroni’s post hoc test. *p < 0.05, **p < 0.005, ***p < 0.0005

Regnase-1 directly binds to stem-loop structures in the 3′-UTRs of target mRNAs to cause their decay (11, 12). To identify target mRNAs of Regnase-1 in chondrocytes, we infected primary-culture chondrocytes with Ad-Regnase-1 or Ad-shRegnase-1 to upregulate and downregulate Regnasse-1, respectively, and conducted microarray analyses. From among the 161 genes found to be downregulated (< 0.6-fold) by Regnase-1 overexpression, only nine were upregulated (> 1.5-fold) by Regnase-1 knockdown (Fig. 6a, Supplementary Table 3). In contrast, of the 67 genes that were upregulated (> 1.5-fold) by Regnase-1 knockdown, 10 genes were downregulated (< 0.7-fold) by Regnase-1 overexpression (Fig. 6a, Supplementary Table 4). The genes that were upregulated by Regnase-1 overexpression or downregulated by Regnase-1 knockdown are listed in Supplementary Tables 5 and 6, respectively.

Of the putative targets of Regnase-1, serum amyloid A3 (SAA3) was the most markedly upregulated by Regnase-1 knockdown and downregulated by Regnase-1 overexpression (Fig. 6b). SAA3 is a member of the SAA protein family of apolipoproteins. Different isoforms of SAA are expressed constitutively (constitutive SAAs) at different levels or in response to inflammatory stimuli (acute-phase SAAs) [28]. We first used the SAA3-3′-UTR reporter plasmid to test whether SAA3 is a bona fide direct target of Regnase-1. We found that the expression of WT Regnase-1 reduced luciferase activity, whereas that of D141N Regnase-1, which lacks RNase activity [13], did not (Fig. 6c). In addition to SAA3, we also found that SAA1, SAA2, and SAA4 also tended to be upregulated by Ad-shRegnase-1 and downregulated by Ad-Regnase-1 (Fig. 6b and d, Supplementary Fig. 5). Among the SAA family members, SAA3 was the most abundant in chondrocytes (Fig. 6d) and the most significantly modulated by Regnase-1 expression (Supplementary Fig. 5). Additionally, marked amounts of SAA were secreted from chondrocytes in response to IL-1β treatment or SAA overexpression (Fig. 6e). We therefore selected SAA3 as a Regnase-1 target for functional analysis in OA pathogenesis.

### SAA3, a Regnase-1 target, does not modulate OA pathogenesis

**Fig. 7:**
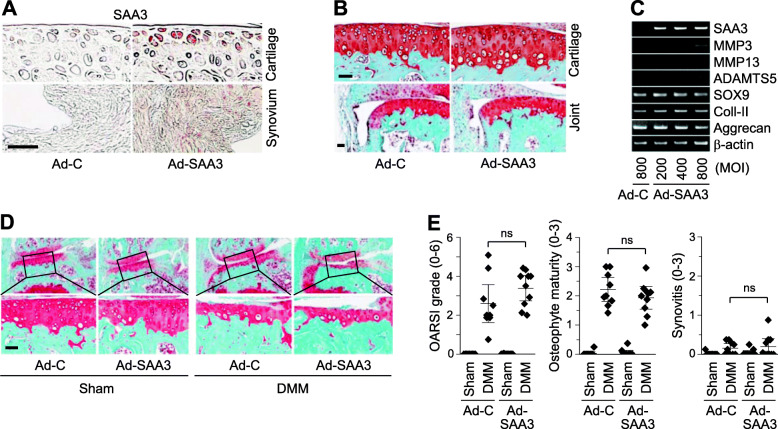
SAA3 does not modulate OA pathogenesis. **a**, **b** Immunohistochemical staining (**a**) and Safranin-O staining (**b**) images from WT mice IA-injected with Ad-C or Ad-SAA3 (*n* = 10 mice per group). Mice were sacrificed at 3 weeks post-injection. **c** Representative RT-PCR images of the indicated molecules in chondrocytes infected with Ad-C or Ad-SAA3 for 36 h (*n* = 6). **d**, **e** Sham- or DMM-operated WT mice were IA-injected with Ad-C or Ad-SAA3. Representative Safranin-O staining images (**d**) and scoring of OARSI grade, osteophyte size, and SBP thickness (**e**) at 6 weeks after the operation (*n* = 10 mice per group). Means ±95% CI with Mann-Whitney *U* test for OARSI grade and means ± s.e.m. with two-tailed *t* test for osteophyte size and SBP thickness. ns, not significant. Scale bar: 50 μm

The function of SAA3 in OA pathogenesis was examined by its overexpression in joint tissues via IA injection of Ad-SAA3 (Fig. 7a). Compared with Ad-C injection, IA injection of Ad-SAA3 in mice did not affect cartilage homeostasis or cause synovial inflammation (Fig. 7b). Consistently, overexpression of SAA3 alone in primary-culture chondrocytes did not modulate the mRNA levels of matrix-degrading enzymes (MMP3, MMP13, and ADAMTS5) or cartilage ECM molecules (type II collagen or aggrecan) (Fig. 7c). Furthermore, IA injection of Ad-SAA3 in sham- or DMM-operated mice did not affect post-traumatic OA manifestations, such as cartilage destruction, osteophyte formation, or synovitis (Fig. 7d, e). Our results collectively indicate that SAA3 overexpression in joint tissues did not cause or modulate OA pathogenesis in mice, suggesting that the chondro-protective functions of Regnase-1 in post-traumatic OA cartilage destruction is mediated by other yet-unidentified target(s) of Regnase-1, rather than SAA3, in chondrocytes.

## Discussion

The post-transcriptional regulation of mRNA stability is an important checkpoint for gene expression, and ribonucleases play a central role in this process. Consequently, abnormalities in ribonucleases have been associated with multiple disease [12, 29, 30]. Indeed, we previously demonstrated that ZFP36L1, an RNA-binding protein that binds to AU-rich elements (AREs) in the 3′-UTRs of its target mRNAs, regulates OA pathogenesis by modulating the mRNA decay of HSP70 family members [27]. Here, we demonstrate that upregulated Regnase-1 in OA chondrocytes protects against post-traumatic OA cartilage destruction by modulating the expression levels of matrix-degrading enzymes. Therefore, our results indicate that upregulated Regnase-1 in OA chondrocytes forms a negative feedback loop to function as a chondro-protective effector molecule in OA cartilage destruction.

One of the key findings of our study is that Regnase-1 decreases the expression levels of matrix-degrading enzymes, such as MMP3, MMP13, and ADAMTS5, in chondrocytes under post-traumatic OA conditions, and thereby suppresses DMM-induced cartilage destruction. Regnase-1 overexpression suppresses the IL-1β- or HIF-2α-induced upregulations of MMP3, whereas KO of Regnase-1 potentiates the upregulations of MMP3, MMP13, and ADAMTS5 with corresponding modulation of cartilage destruction. Regnase-1 causes decay of target mRNAs by specifically binding to UAU and UGU loops of the 3′-UTR located more than 20 nucleotides away from the stop codon [31]. According to RegRNA2.0 analysis [32], the mRNAs of mouse MMP3, MMP13, and ADAMTS5 have loops that meet these criteria. However, unlike the regulation of these enzymes in the presence of catabolic stimuli (i.e., IL-1β and HIF-2α), overexpression or knockdown of Regnase-1 in unstimulated chondrocytes did not markedly modulate the expression levels of these enzymes (Supplementary Tables 3–6). Therefore, our results indicate that Regnase-1 regulates the expression levels of matrix-degrading enzymes via indirect regulatory mechanisms, rather than by direct targeting.

Regnase-1 regulates a variety of different target mRNAs in different cell types [11–15]. The mRNA of IL-6 has been identified as a Regnase-1 target in multiple cell types, including macrophages [20, 33], T cells [13, 14], and chondrocytes [16]. In unstimulated human OA chondrocytes, overexpression of Regnase-1 decreases the IL-6 mRNA level whereas expression of D141N mutant Reganse-1 (lacking RNase activity) does not [16]; this suggested that the IL-6 mRNA is a target of Reganse-1, although this was not confirmed in the previous study. Because IL-6 plays a critical role in OA pathogenesis [19], we initially speculated that Regnase-1 may exert its effects by modulating the IL-6 mRNA. However, similar to our observations regarding matrix-degrading enzymes, we found that overexpression or knockdown of Regnase-1 in unstimulated chondrocytes did not modulate the mRNA expression of IL-6 (Supplementary Tables 3–6). This suggests that IL-6 mRNA is not a direct target of Regnase-1 in mouse articular chondrocytes and does not play a role in the ability of Regnase-1 to regulate post-traumatic OA cartilage destruction. This finding is consistent with previous report indicating that the targets of Regnase-1 vary dramatically among different cells and conditions [11, 34]. It has been suggested that the process of Regnase-1 recognition is likely to be determined by multiple factors and distinct layers of regulation that may differ among cell types [11, 34].

To identify mediator(s) through which Regnase-1 regulates OA cartilage destruction, we tried to identify Regnase-1 target mRNAs via microarray analysis in chondrocytes with overexpression or knockdown of Regnase-1. We found that SAA family members met the criteria for Regnase-1 targets: They were downregulated by Regnase-1 overexpression and upregulated by Regenase-1 knockdown and contain UAU and UGU loops in their 3′-UTRs. SAA1 and SAA2 are classic acute phase response serum proteins in humans and mice; SAA3 is a pseudogene in humans that is transcribed but not translated; and SAA4 is expressed constitutively in humans [28]. The SAAs have been suggested to play roles in inflammatory diseases, such as atherosclerosis, rheumatoid arthritis, and chronic inflammatory bowel disease and may function in cholesterol transport [35]. SAAs have also been reported to induce the expression of pro-inflammatory genes via NF-κB pathways [36], and recombinant SAA3 was reported to induce MMP13 expression in rabbit articular chondrocytes [37]. Here, we characterized the possible functions of SAA3 in the Regnase-1-mediated regulation of OA pathogenesis, as it was the most abundantly expressed and significantly modulated SAA isoform in our system. However, our results revealed that overexpression of SAA3 in mouse articular chondrocytes does not modulate the expression of matrix-degrading enzymes or cartilage ECM molecules, and OA cartilage destruction is not modulated by the overexpression of SAA3 in mouse joint tissues. Therefore, it is likely that yet-unidentified Regnase-1 targets other than SAA3 may mediate the chondro-protective functions of Regnase-1 during OA pathogenesis. However, although we confirmed the expression of SAA3 protein by immunohistochemical staining in cartilage and synovial tissues, it remains possible that the levels of overexpressed SAA3 protein may not have been sufficient to cause any catabolic effects in joint tissues.

## Conclusions

The present results suggest that upregulated Regnase-1 in OA chondrocytes may functions as a chondro-protective effector molecule during OA pathogenesis by forming a negative feedback loop of catabolic signals, such as the expression of matrix-degrading enzymes, in OA chondrocytes.

## Supplementary Information

**Additional file 1: Supplementary Fig. 1.** Upregulation of Regnase-1 in chondrocytes and fibroblast-like synoviocytes (FLS) treated with OA-associated catabolic factors. **A**–**C** RT-PCR analysis of ZC3H12 family members in chondrocytes treated with IL-1 β (**A**), infected with Ad-HIF-2α or Ad-ZIP8 (**B**), or infected with Ad-Regnase-1 (**C**). **D** and **E** RT-PCR (**D**) and qRT-PCR (**E**) analysis of ZC3H12 family members in FLS treated with IL-1β or infected with Ad-HIF-2α or Ad-ZIP8. **F** RT-PCR analysis of the indicate molecules in FLS infected with Ad-Regnase-1 or AdshRegnase-1. Images are representative of the results obtained from five independent primary cultures of chondrocytes or FLS. Values are means ± s.e.m. and one-way ANOVA with Bonferroni’s post hoc test. *p < 0.05, ***p < 0.0005. ns, not significant. **Supplementary Fig. 2.** Knockdown of Regnase-1 alone in joint tissues is not sufficient to cause OA-like changes in mice. **A** Representative Safranin-O staining images of joint sections from mice IA-injected with Ad-shScramble (Ad-shC), Ad-shRegnase-1, or Ad-HIF-2α (*n* = 10 mice per group). IA injection of Ad-HIF-2α was used as a positive control. **B** and **C** Representative RT-PCR images (*n* = 4) of the indicated molecules in primary-culture chondrocytes infected with Ad-shControl (AdshC) or Ad-shRegnase-1. **D** Representative immunohistochemical staining images (*n* ≥ 5 mice per group) of the indicated molecules in cartilage and synovium of sham- or DMM-operated mice injected with As-shScramble or Ad-shRegnase-1. Scale bar: 50 μm. **Supplementary Fig. 3**. Characterization of Regnase-1 knockout mice. **A** A 68-bp deletion in exon 2 of the Regnase-1 gene (*Zc3h12a*) was used to generate *Zc3h12a−/−* mice. **B** Genotypes and mRNA levels of ZC3H12A family members in *Zc3h12a* homozygous (−/−) KO mice, heterozygous (+/−) mice, and their WT (+/+) littermates. **C** Skeletal staining of E18.5 embryos of *Zc3h12a* homozygous (−/−) KO mice, heterozygous (+/−) KO mice, and their WT (+/+) littermates. **D** Representative immunohistochemical staining images (*n* ≥ 5 mice per group) of the indicated molecules in cartilage or synovium of sham- or DMM-operated WT mice and *Zc3h12a+/−* mice. Scale bar: 50 μm. **Supplementary Fig. 4.** Regnase-1 does not modulate apoptosis of chondrocytes. **A** Representative RT-PCR images of the indicated molecules in chondrocytes infected with Ad-Regnase-1 or Ad-shRegnase-1 (*n* = 3). SNP (sodium nitroprusside) was used as a positive control. **B** and **C** Representative images of TUNEL assays (left panels) and quantitation of apoptotic chondrocytes (right panels) in cartilage sections of sham- or DMM-operated mice IA injected with Ad-Regnase-1 (**B**) or Ad-shRegnase-1 (**C**) (*n* ≥ 4 mice per group). Values are means ± s.e.m. and one-way ANOVA with Bonferroni’s post hoc test. *p < 0.05, **p < 0.005, ***p < 0.0005. ns, not significant. AC, articular cartilage; CC, calcified cartilage. **Supplementary Fig. 5**. Regnase-1 modulates the expression levels of SAA family members in chondrocytes. **A** and **B** qRT-PCR analysis (*n* = 6) of SAA family members (SAA1, SAA2, SAA3, and SAA4) in chondrocytes infected with 800 MOI of control adenovirus (Ad-C or Ad-shC) or the indicated MOI of Ad-Regnase-1 (**A**) and Ad-shRegnase-1 (**B**) for 36 h. Values are means ± s.e.m. and one-way ANOVA with Bonferroni’s post hoc test. *p < 0.05, **p < 0.005, ***p < 0.0005. ns, not significant. **Supplementary Table 1**. PCR primers and conditions. **Supplementary Table 2**. mRNA levels of RNases in chondrocytes treated with IL-1β (1 ng/ml, 36 h) or infected with 800 MOI of Ad-HIF-2α or Ad-ZIP8 (36 h). **Supplementary Table 3**. List of down-regulated genes (< 0.6-fold) following overexpression of Regnase-1 via Ad-Regnase-1 infection in chondrocytes. **Supplementary Table 4**. List of up-regulated genes (> 1.5-fold) following knock-down of Regnase-1 via Ad-shRegnase-1A infection in chondrocytes. **Supplementary Table 5**. List of up-regulated genes (> 2.0-fold) following overexpression of Regnase-1 via Ad-Regnase-1 infection in chondrocytes. **Supplementary Table 6**. List of down-regulated genes (< 0.7-fold) following knock-down of Regnase-1 via AdshRegnase-1 infection in chondrocytes.

## Data Availability

The data supporting the conclusions of this article are included within the article.

## Abbreviations

ARE: AU-rich elements
ECM: Extracellular matrix
IL: Interleukin
HIF: Hypoxia-inducible factor
KO: Knock-out
MMP: Matrix metalloproteinase
OA: Osteoarthritis
SAA: Serum amyloid A
WT: Wild-type
