# Supplementary material for: Upregulated endonuclease Regnase-1 suppresses osteoarthritis by forming a negative feedback loop of catabolic signaling in chondrocytes
Source: Arthritis Res Ther. 2021 Apr 14;23:114. doi: 10.1186/s13075-021-02485-z (PMC8045248; doi:10.1186/s13075-021-02485-z)
Supplement: Supplementary file 1 — Additional file 1: Supplementary Fig. 1. Upregulation of Regnase-1 in chondrocytes and fibroblast-like synoviocytes (FLS) treated with OA-associated catabolic factors. A–C RT-PCR analysis of ZC3H12 family members in chondrocytes treated with IL-1 β (A), infected with Ad-HIF-2α or Ad-ZIP8 (B), or infected with Ad-Regnase-1 (C). D and E RT-PCR (D) and qRT-PCR (E) analysis of ZC3H12 family members in FLS treated with IL-1β or infected with Ad-HIF-2α or Ad-ZIP8. F RT-PCR analysis of the indicate molecules in FLS infected with Ad-Regnase-1 or AdshRegnase-1. Images are representative of the results obtained from five independent primary cultures of chondrocytes or FLS. Values are means ± s.e.m. and one-way ANOVA with Bonferroni’s post hoc test. *p < 0.05, ***p < 0.0005. ns, not significant. Supplementary Fig. 2. Knockdown of Regnase-1 alone in joint tissues is not sufficient to cause OA-like changes in mice. A Representative Safranin-O staining images of joint sections from mice IA-injected with Ad-shScramble (Ad-shC), Ad-shRegnase-1, or Ad-HIF-2α (n = 10 mice per group). IA injection of Ad-HIF-2α was used as a positive control. B and C Representative RT-PCR images (n = 4) of the indicated molecules in primary-culture chondrocytes infected with Ad-shControl (AdshC) or Ad-shRegnase-1. D Representative immunohistochemical staining images (n ≥ 5 mice per group) of the indicated molecules in cartilage and synovium of sham- or DMM-operated mice injected with As-shScramble or Ad-shRegnase-1. Scale bar: 50 μm. Supplementary Fig. 3. Characterization of Regnase-1 knockout mice. A A 68-bp deletion in exon 2 of the Regnase-1 gene (Zc3h12a) was used to generate Zc3h12a−/− mice. B Genotypes and mRNA levels of ZC3H12A family members in Zc3h12a homozygous (−/−) KO mice, heterozygous (+/−) mice, and their WT (+/+) littermates. C Skeletal staining of E18.5 embryos of Zc3h12a homozygous (−/−) KO mice, heterozygous (+/−) KO mice, and their WT (+/+) littermates. D Representative immunohistoc [file 13075_2021_2485_MOESM1_ESM.pdf]

Supplementary Materials

Up-regulated endonuclease Regnase-1 suppresses osteoarthritis by forming a negative feedback loop of catabolic signaling in chondrocytes

Jeong-In Yang and Jang-Soo Chun

Supplementary Figure 1-5

Supplementary Table 1-6

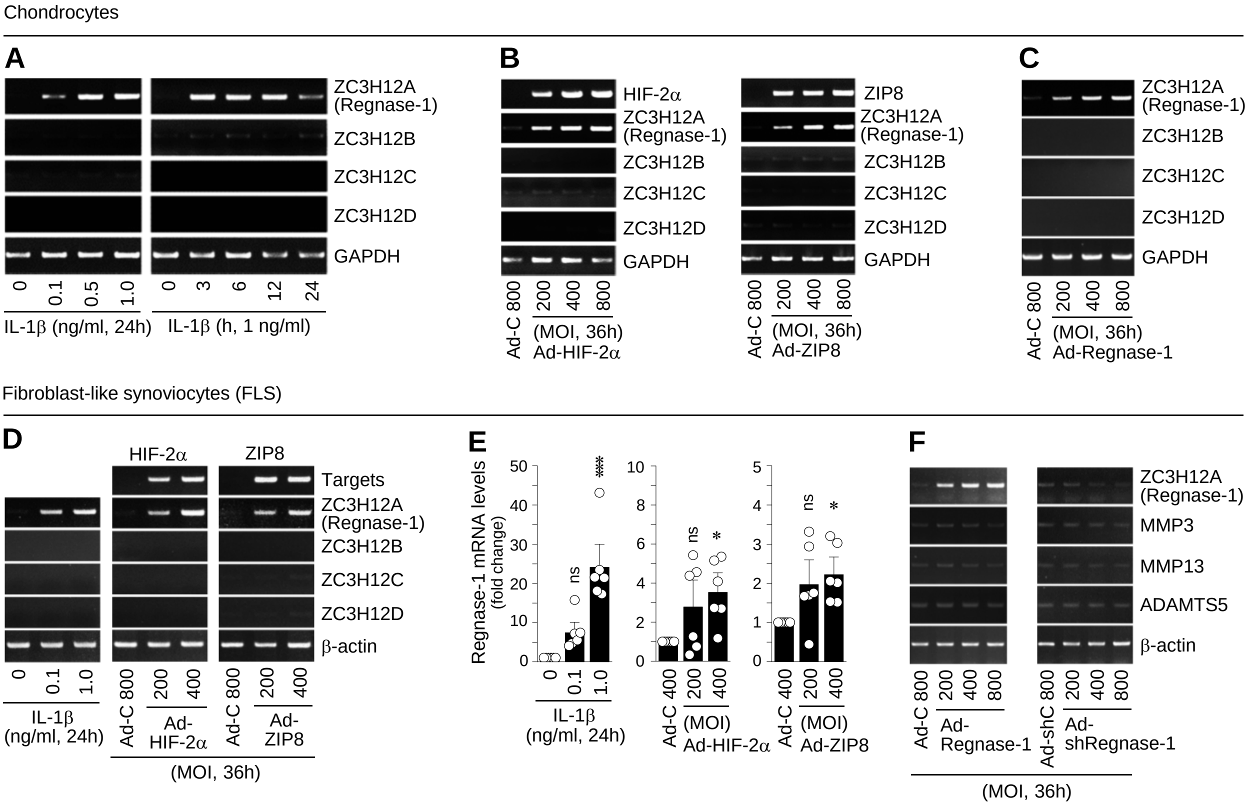

Supplementary Fig. 1. Upregulation of Regnase-1 in chondrocytes and fibroblast-like synoviocytes (FLS) treated with OA-associated catabolic factors. **A**–**C** RT-PCR analysis of ZC3H12 family members in chondrocytes treated with IL-1β (**A**), infected with Ad-HIF-2α or Ad-ZIP8 (**B**), or infected with Ad-Regnase-1 (**C**). **D** and **E** RT-PCR (**D**) and qRT-PCR (**E**) analysis of ZC3H12 family members in FLS treated with IL-1β or infected with Ad-HIF-2α or Ad-ZIP8. **F** RT-PCR analysis of the indicate molecules in FLS infected with Ad-Regnase-1 or Ad-shRegnase-1. Images are representative of the results obtained from five independent primary cultures of chondrocytes or FLS. Values are means ± s.e.m. and one-way ANOVA with Bonferroni's *post hoc* test. *p < 0.05, ***p < 0.0005. ns, not significant.

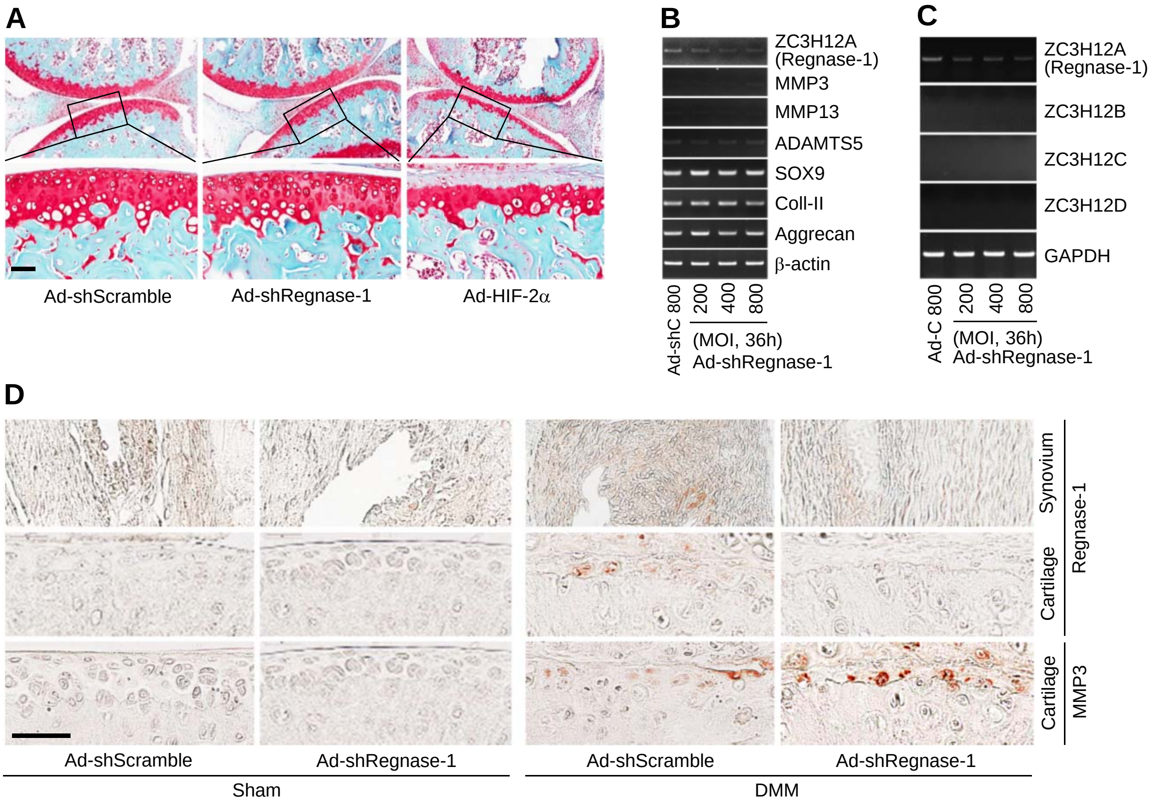

Supplementary Fig. 2. Knockdown of Regnase-1 alone in joint tissues is not sufficient to cause OA-like changes in mice. **A** Representative Safranin-O staining images of joint sections from mice IA-injected with Ad-shScramble (Ad-shC), Ad-shRegnase-1, or Ad-HIF-2α (n = 10 mice per group). IA injection of Ad-HIF-2α was used as a positive control. **B** and **C** Representative RT-PCR images (n = 4) of the indicated molecules in primary-culture chondrocytes infected with Ad-shControl (Ad-shC) or Ad-shRegnase-1. **D** Representative immunohistochemical staining images ($n \geq 5$ mice per group) of the indicated molecules in cartilage and synovium of sham- or DMM-operated mice injected with As-shScramble or Ad-shRegnase-1. Scale bar: 50 µm.

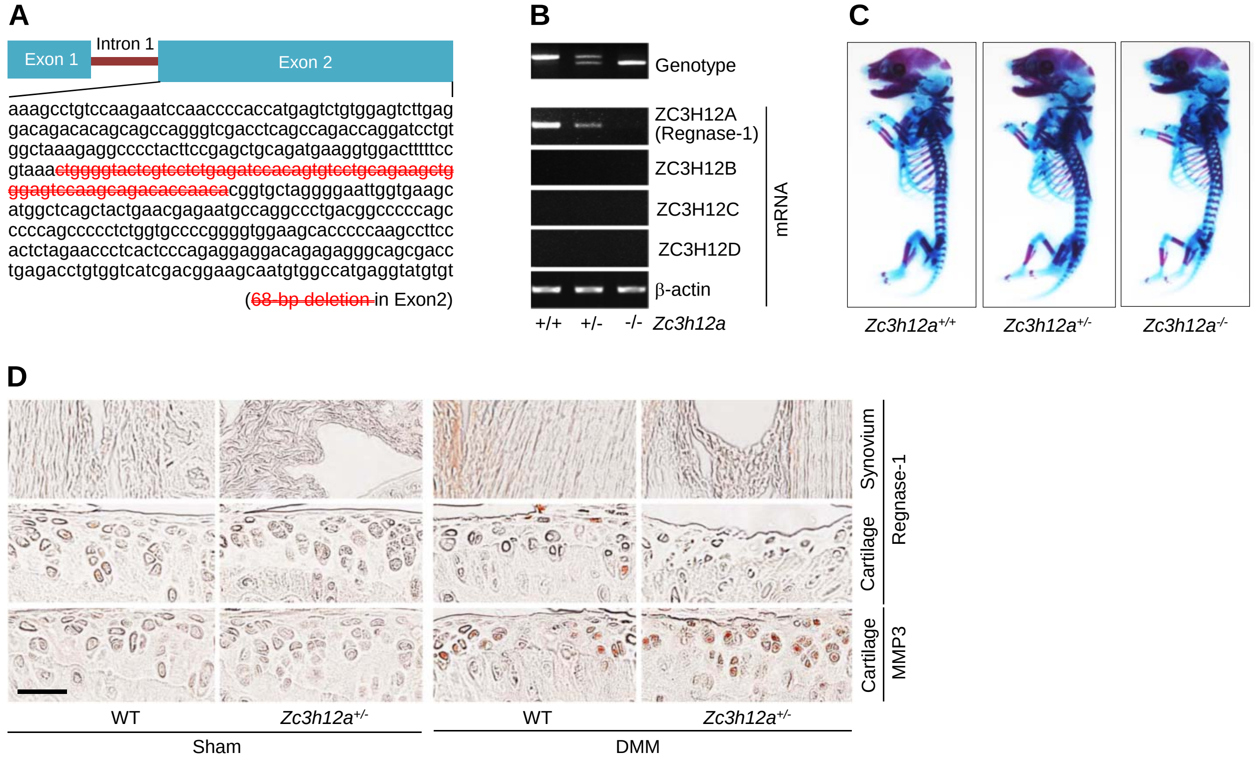

Supplementary Fig. 3. Characterization of Regnase-1 knockout mice. **A** A 68-bp deletion in exon 2 of the Regnase-1 gene (*Zc3h12a*) was used to generate *Zc3h12a*$^{-/-}$ mice. **B** Genotypes and mRNA levels of ZC3H12A family members in *Zc3h12a* homozygous (-/-) KO mice, heterozygous (+/-) mice, and their WT (+/+) littermates. **C** Skeletal staining of E18.5 embryos of *Zc3h12a* homozygous (-/-) KO mice, heterozygous (+/-) KO mice, and their WT (+/+) littermates. **D** Representative immunohistochemical staining images ($n \geq 5$ mice per group) of the indicated molecules in cartilage or synovium of sham- or DMM-operated WT mice and *Zc3h12a*$^{+/-}$ mice. Scale bar: 50 μm.

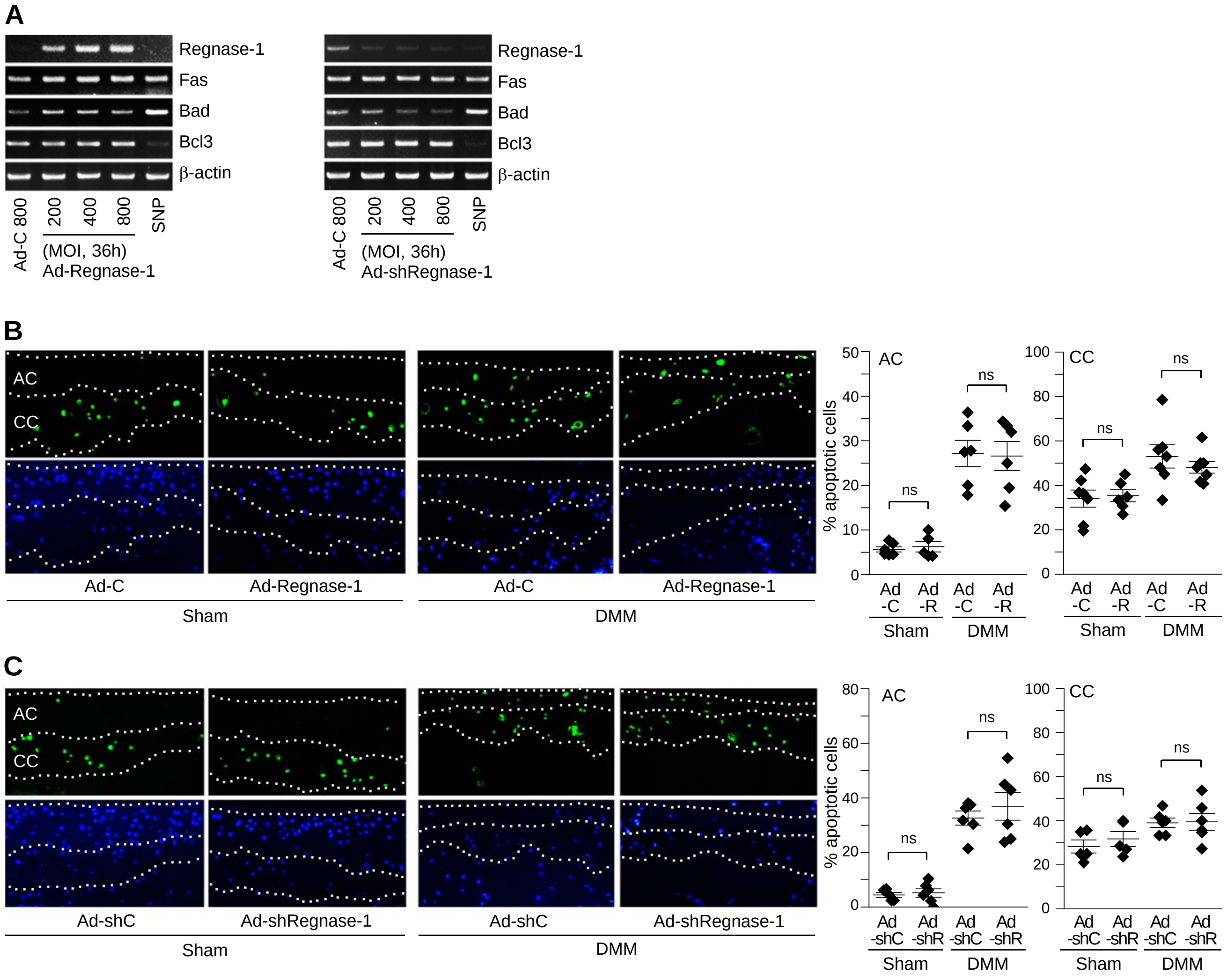

Supplementary Fig. 4. Regnase-1 does not modulate apoptosis of chondrocytes. **A** Representative RT-PCR images of the indicated molecules in chondrocytes infected with Ad-Regnase-1 or Ad-shRegnase-1 (n = 3). SNP (sodium nitroprusside) was used as a positive control. **B** and **C** Representative images of TUNEL assays (left panels) and quantitation of apoptotic chondrocytes (right panels) in cartilage sections of sham- or DMM-operated mice IA injected with Ad-Regnase-1 (**B**) or Ad-shRegnase-1 (**C**) ($n \geq 4$ mice per group). Values are means ± s.e.m. and one-way ANOVA with Bonferroni's *post hoc* test. *p < 0.05, **p < 0.005, ***p < 0.0005. ns, not significant. AC, articular cartilage; CC, calcified cartilage.

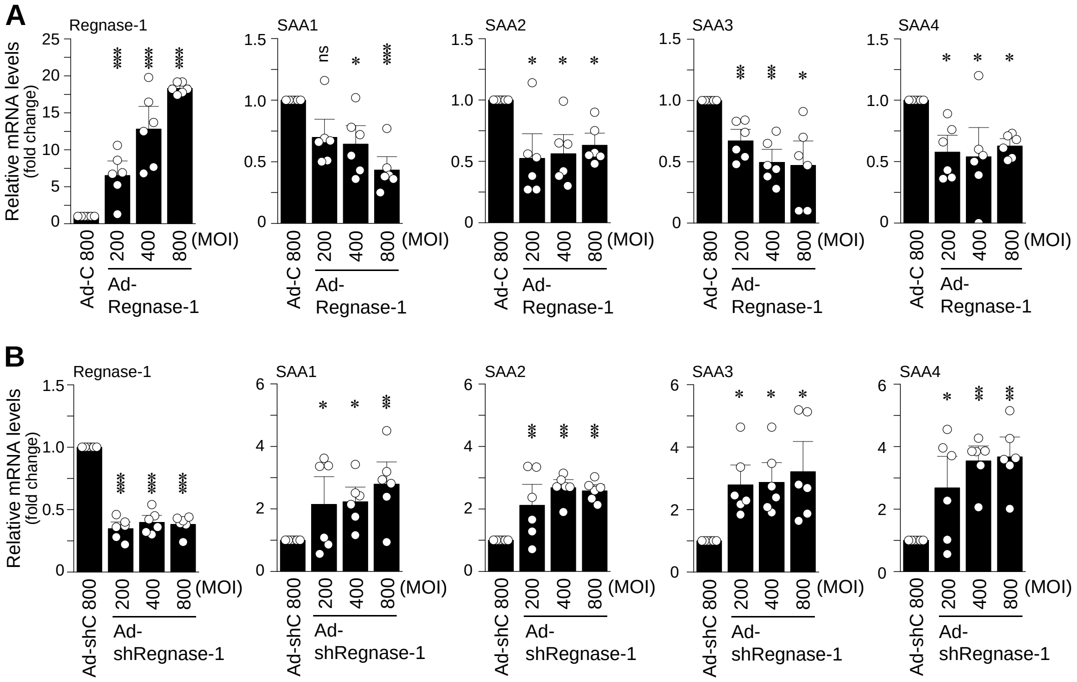

Supplementary Fig. 5. Regnase-1 modulates the expression levels of SAA family members in chondrocytes. **A** and **B** qRT-PCR analysis (n = 6) of SAA family members (SAA1, SAA2, SAA3, and SAA4) in chondrocytes infected with 800 MOI of control adenovirus (Ad-C or Ad-shC) or the indicated MOI of Ad-Regnase-1 (**A**) and Ad-shRegnase-1 (**B**) for 36 hours. Values are means ± s.e.m. and one-way ANOVA with Bonferroni's *post hoc* test. *p < 0.05, **p < 0.005, ***p < 0.0005. ns, not significant.

Supplementary Table 1. PCR primers and conditions.

Genes	Strand	Primer sequences	Size (bp)	AT (°C)	Origin
Acan (Aggrecan)	S<br>AS	5′-CTGTCTTTGTCACCCACACATG-3′<br>5′-GAAGACGACATCACCATCCAG-3′	581	58	Mouse
Adamts5	S<br>AS	5′-GCCATTGTAATAACCCTGCACC-3′<br>5′-TCAGTCCCATCCGTAACCTTTG-3′	292	58	Mouse
Actb (β-actin)	S<br>AS	5′-ATATCGCTGCGCTGGTCGTC-3′<br>5′-AGGATGGCGTGAGGGAGAGC-3	517	58	Mouse
Bad	S<br>AS	5′-AGGGATGGAGGAGGAGCTTA -3′<br>5′-TAGAGTTCCGGGATGTGGAG -3′	280	60	Mouse
Bcl3	S<br>AS	5′-GTGGAGAACAACAGCCTGAACATGG-3′<br>5′-GCAGGAAGGCAGGTGTAGATGTTG-3′	445	60	Mouse
Col2a1	S<br>AS	5′-CACACTGGTAAGTGGGGCAAGACCG-3′<br>5′-GGATTGTGTTGTTTCAGGGTTCGGG-3′	173	57	Mouse
Epas1 (HIF-2α)	S<br>AS	5′-CGAGAAGAACGACGTGGTGTTC-3′<br>5′-GTGAAGGCTGGCAGGCTCC-3′	333	64	Mouse
Fas	S<br>AS	5′-CTCTGGTGCTTGCTGGCTCACAGTTAAG-3′<br>5′-TTTCAGGTTGGCATGGTTGACAGC-3′	162	56	Mouse
Mmp13	S<br>AS	5′-TGATGGACCTTCTGGTCTTCTGG-3′<br>5′-CATCCACATGGTTGGGAAGTTCT-3′	473	58	Mouse
Mmp3	S<br>AS	5′-AGGGATGATGATGCTGGTATGG-3′<br>5′-CCATGTTCTCCAACTGCAAAGG-3′	434	58	Mouse
Saa1	S<br>AS	5′-TCCTCCTCAAGCAGTTACTACTGCAA-3′<br>5′-ATGAAGGAAGCTAACTGGAAAAACTC-3′	423	60	Mouse
Saa2	S<br>AS	5′-TCCTCCTCAAGCAGTTACTACTGCTC-3′<br>5′-ATGAAGGAAGCTGGCTGGAAAGATGG-3′	420	60	Mouse
Saa3	S<br>AS	5′-GCCACCATGAAGCCTTCCATTGCCATCATT-3′<br>5′-TCAGTATCTTTTAGGCAGGCCAGCAG-3′	375	60	Mouse
Saa4	S<br>AS	5′-GAGGTCTTGCTCGTGATTCACT-3′<br>5′-TTTCTGGGTAGCCTGCAGGGTT-3′	373	66	Mouse
Slc39a8 (ZIP8)	S<br>AS	5′-GAACAATTGCCTGGATGATCACGC-3′<br>5′-AAGCCGGTTAACATCCCTGCATTC-3′	430	58	Mouse
Sox9	S<br>AS	5′-CACTGGCAGTTACGGCATCAG-3′<br>5′-CATGTAAGTGAAGGTGGAGTAGAGC-3′	457	61	Mouse
Zc3h12a	S<br>AS	5′-AGATGTCAGAATTATGGGGCGTGC-3′<br>5′-GCGTCTCATTACAGCTTCTACCAGG-3′	389	64	Mouse
Zc3h12b	S<br>AS	5′-GAGGGTGGTCTGCTATGATGA-3′<br>5′-GAAATTCTCAAGGCTCGGACC-3′	217	60	Mouse
Zc3h12c	S<br>AS	5′-CTCAGGATCAAAGACCACAGG-3′<br>5′-AATATCCAACGTCAACAGGGG-3′	120	60	Mouse
Zc3h12d	S<br>AS	5′-GGCGATCTCGGGTCTCCAAT-3′<br>5′-GGTTGTGCTGACTCCGAAGG-3′	212	60	Mouse

AT, annealing temperature; S, sense; AS, antise

Supplementary Table 2. mRNA levels of RNases in chondrocytes treated with IL-1β (1 ng/ml, 36 h) or infected with 800 MOI of Ad-HIF-2α or Ad-ZIP8 (36 h).

Gene symbol	Definition	Fold change		
		IL-1β	Ad-HIF-2α	Ad-ZIP8
Endonuclease				
Ago2	Argonaute RISC catalytic subunit 2	1.069 ± 0.08	1.143 ± 0.13	1.033 ± 0.12
Ang2	Angiogenin, ribonuclease A family, member 2	1.219 ± 0.20	1.074 ± 0.43	1.280 ± 0.76
Ang4	Angiogenin, ribonuclease A family, member 4	0.757 ± 0.65	0.991 ± 0.63	0.876 ± 0.30
Ang5	Angiogenin, ribonuclease A family, member 5	0.973 ± 0.14	1.025 ± 0.31	1.121 ± 0.31
Ang6	Angiogenin, ribonuclease A family, member 6	1.117 ± 0.24	0.987 ± 0.26	0.919 ± 0.32
Apex1	Apurinic/apyrimidinic endonuclease 1	0.818 ± 0.18	0.985 ± 0.07	1.149 ± 0.06
Apex2	Apurinic/apyrimidinic endonuclease 2	0.898 ± 0.08	0.924 ± 0.11	0.871 ± 0.15
Cpsf3	Cleavage and polyadenylation specificity factor 3	0.868 ± 0.12	0.881 ± 0.10	0.903 ± 0.13
Dclre1c	DNA cross-link repair 1C, PSO2 homolog	1.363 ± 0.22	1.052 ± 0.23	1.160 ± 0.18
Dicer1	ElaC homolog 2 (E. coli)	0.600 ± 0.22	0.792 ± 0.15	0.779 ± 0.16
Ear1	Eosinophil-associated, ribonuclease A family, member 1	0.919 ± 0.49	1.156 ± 0.59	1.050 ± 0.65
Ear10	Eosinophil-associated, ribonuclease A family, member 10	0.917 ± 0.16	0.958 ± 0.32	1.091 ± 0.40
Ear11	Eosinophil-associated, ribonuclease A family, member 11	-	1.286 ± 0.21	1.190 ± 0.33
Ear2	Eosinophil-associated, ribonuclease A family, member 2	1.055 ± 0.21	1.031 ± 0.20	1.071 ± 0.22
Ear4	Eosinophil-associated, ribonuclease A family, member 4	-	0.950 ± 0.37	0.914 ± 0.29
Ear5	Eosinophil-associated, ribonuclease A family, member 5	-	0.977 ± 0.21	0.975 ± 0.36
Ear7	Eosinophil-associated, ribonuclease A family, member 7	-	1.125 ± 0.40	1.156 ± 0.41
Elac1	Zinc phosphodiesterase ELAC protein 1	1.117 ± 0.16	1.101 ± 0.15	1.002 ± 0.20
Elac2	Zinc phosphodiesterase ELAC protein 2	0.779 ± 0.12	0.829 ± 0.05	0.830 ± 0.07
Eme1	Essential meiotic endonuclease 1 homolog 1	0.853 ± 0.27	1.142 ± 0.35	1.017 ± 0.24
Endod1	Endonuclease domain containing 1	0.991 ± 0.11	1.068 ± 0.14	1.049 ± 0.17
Endog	Endonuclease G	0.769 ± 0.16	0.924 ± 0.19	0.873 ± 0.18
Endou	Endonuclease, polyU-specific	1.050 ± 0.21	1.097 ± 0.15	0.994 ± 0.25
Endov	Inosine-specific endoribonuclease	1.017 ± 0.04	0.839 ± 0.17	0.893 ± 0.17
Ern2	Endoplasmic reticulum (ER) to nucleus signaling 2	-	1.006 ± 0.21	1.001 ± 0.28
Exo1	Exonuclease 1	0.496 ± 0.26	1.687 ± 0.24	1.944 ± 0.24
Exog	Exo/endonuclease G	0.617 ± 0.20	0.773 ± 0.14	0.757 ± 0.17
Fan1	FANCD2/FANCI-associated nuclease 1	0.900 ± 0.35	0.938 ± 0.19	0.888 ± 0.26
Fen1	Flap structure specific endonuclease 1	0.622 ± 0.32	1.216 ± 0.16	1.163 ± 0.13
Hrsp12	Heat-responsive protein 12	1.250 ± 0.13	1.019 ± 0.16	1.000 ± 0.13
Mre11a	Meiotic recombination 11 homolog A (S. cerevisiae)	0.735 ± 0.23	0.927 ± 0.19	0.950 ± 0.12
Mrpl44	Mitochondrial ribosomal protein L44	1.009 ± 0.09	0.880 ± 0.13	0.975 ± 0.16
Mus81	MUS81 endonuclease homolog (yeast)	1.049 ± 0.16	1.013 ± 0.15	0.920 ± 0.18
Pelo	Pelota MRNA surveillance and ribosome rescue factor	0.906 ± 0.06	0.869 ± 0.11	0.863 ± 0.14
Pld6	Phospholipase D family, member 6	0.946 ± 0.18	0.971 ± 0.19	1.121 ± 0.18

Rnase4	Ribonuclease, RNase A family 4	-	0.907 ± 0.39	1.431 ± 0.36
Rnase6	Ribonuclease, RNase A family, 6	0.966 ± 0.40	1.001 ± 0.19	1.042 ± 0.13
Rnaseh1	Ribonuclease H1	-	0.904 ± 0.07	0.934 ± 0.08
Rnasel	Ribonuclease L	1.597 ± 0.31	1.337 ± 0.27	1.363 ± 0.32
Rnaset2b	Ribonuclease T2	0.827 ± 0.33	0.866 ± 0.17	0.886 ± 0.19
Slx4	SLX4 structure-specific endonuclease subunit	1.008 ± 0.08	0.957 ± 0.17	0.990 ± 0.17
Tsen34	tRNA splicing endonuclease 34 homolog	0.869 ± 0.18	1.138 ± 0.11	1.052 ± 0.13
Tsn	Translin RNase	0.894 ± 0.18	0.978 ± 0.14	1.016 ± 0.10
Zc3h12a	Zinc finger CCCH type containing 12A	7.781 ± 0.14	4.744 ± 0.47	3.949 ± 0.44
Zc3h12b	Zinc finger CCCH type containing 12B	1.323 ± 0.37	0.940 ± 0.34	1.025 ± 0.29
Zc3h12c	Zinc finger CCCH type containing 12C	0.948 ± 0.20	1.208 ± 0.16	1.163 ± 0.19
Exonucleases				
Apex1	apurinic/apyrimidinic endonuclease 1	0.818 ± 0.18	0.985 ± 0.07	1.149 ± 0.06
Apex2	apurinic/apyrimidinic endonuclease 2	0.898 ± 0.08	0.924 ± 0.11	0.871 ± 0.15
Cnot6	CCR4-NOT transcription complex, subunit 6	0.966 ± 0.24	0.968 ± 0.17	0.962 ± 0.14
Cnot6l	CCR4-NOT transcription complex, subunit 6-like	1.006 ± 0.12	0.975 ± 0.10	0.941 ± 0.13
Cnot7	CCR4-NOT transcription complex, subunit 7	1.279 ± 0.17	0.918 ± 0.07	0.892 ± 0.08
Cnot8	CCR4-NOT transcription complex, subunit 8	0.881 ± 0.17	0.821 ± 0.16	0.807 ± 0.13
Cpsf3	cleavage and polyadenylation specificity factor 3	0.868 ± 0.12	0.881 ± 0.10	0.903 ± 0.13
Ddx1	DEAD (Asp-Glu-Ala-Asp) box polypeptide 1	0.798 ± 0.08	0.938 ± 0.10	0.909 ± 0.09
Dis3	DIS3 mitotic control homolog (S. cerevisiae)	0.578 ± 0.02	0.778 ± 0.25	0.831 ± 0.18
Dis3l	DIS3 mitotic control homolog (S. cerevisiae)-like	0.857 ± 0.09	0.959 ± 0.23	0.979 ± 0.32
Dis3l2	DIS3 mitotic control homolog (S. cerevisiae)-like 2	1.104 ± 0.17	1.076 ± 0.18	1.051 ± 0.24
Dxo	decapping exoribonuclease	1.042 ± 0.16	1.002 ± 0.17	0.955 ± 0.26
Eri1	exoribonuclease 1	0.707 ± 0.25	1.100 ± 0.15	1.128 ± 0.17
Eri2	exoribonuclease 2	1.043 ± 0.26	1.142 ± 0.20	1.135 ± 0.23
Eri3	exoribonuclease 3	0.904 ± 0.24	0.913 ± 0.28	0.900 ± 0.29
Exd1	exonuclease 3-5 domain containing 1	1.190 ± 0.18	1.157 ± 0.19	1.274 ± 0.27
Exd2	exonuclease 3-5 domain containing 2	0.939 ± 0.13	0.932 ± 0.38	0.955 ± 0.39
Exosc3	exosome component 3	0.932 ± 0.18	0.990 ± 0.22	0.979 ± 0.17
Exosc9	exosome component 9	0.862 ± 0.08	0.897 ± 0.12	0.921 ± 0.08
Isg20	interferon-stimulated protein	0.917 ± 0.09	1.072 ± 0.08	1.040 ± 0.10
Isg20l2	interferon stimulated exonuclease gene 20-like 2	0.827 ± 0.12	0.953 ± 0.04	0.990 ± 0.06
Pan2	PAN2 polyA specific ribonuclease subunit homolog	0.903 ± 0.08	1.030 ± 0.14	0.955 ± 0.23
Parn	poly(A)-specific ribonuclease (deadenylation nuclease)	0.858 ± 0.16	0.905 ± 0.11	0.992 ± 0.09
Pnpt1	phosphodiesterase 12	0.778 ± 0.34	0.878 ± 0.13	0.944 ± 0.11
Rexo1	REX1, RNA exonuclease 1 homolog (S. cerevisiae)	0.934 ± 0.09	0.986 ± 0.19	0.925 ± 0.19
Rexo2	REX2, RNA exonuclease 2 homolog (S. cerevisiae)	0.922 ± 0.13	1.066 ± 0.11	1.044 ± 0.08
Rexo4	REX4, RNA exonuclease 4 homolog (S. cerevisiae)	0.970 ± 0.16	0.934 ± 0.05	0.942 ± 0.09
Xrn1	5-3 exoribonuclease 1	1.206 ± 0.19	1.025 ± 0.22	0.994 ± 0.17
Xrn2	5-3 exoribonuclease 2	0.894 ± 0.09	1.047 ± 0.13	1.039 ± 0.11

Supplementary Table 3. List of down-regulated genes (< 0.6-fold) following overexpression of Regnase-1 via Ad-Regnase-1 infection in chondrocytes.

Gene symbol	Definition	Fold change				
		Ad-Regnase-1	Ad-shRegnase-1	IL-1β	Ad-HIF-2α	Ad-ZIP8
Fgf21	Fibroblast growth factor 21	0.248 ± 0.56	1.004 ± 0.48	0.128 ± 0.30	0.407 ± 0.23	0.509 ± 0.27
Abi3bp	ABI gene family, member 3 (NESH) binding protein	0.303 ± 0.28	1.088 ± 0.24	1.110 ± 0.25	0.690 ± 0.21	0.917 ± 0.26
Mir434	MicroRNA 434	0.338 ± 0.43	1.331 ± 0.75	0.242 ± 0.29	0.357 ± 0.46	0.471 ± 0.50
Pycr1	Pyrroline-5-carboxylate reductase 1	0.361 ± 0.28	0.857 ± 0.28	0.412 ± 0.17	0.494 ± 0.19	0.573 ± 0.19
Gm12829	Predicted gene 12829	0.362 ± 0.26	1.206 ± 0.51	0.411 ± 0.44	0.423 ± 0.30	0.715 ± 0.48
Cnn1	Calponin 1	0.381 ± 0.42	0.938 ± 0.25	0.856 ± 0.23	0.345 ± 0.31	0.415 ± 0.28
Npr3	Natriuretic peptide receptor 3	0.387 ± 0.22	0.951 ± 0.13	0.262 ± 0.28	0.512 ± 0.17	0.529 ± 0.35
Chrdl1	Chordin-like 1	0.387 ± 0.34	1.016 ± 0.36	0.549 ± 0.27	0.305 ± 0.41	0.426 ± 0.23
Igfbp5	Insulin-like growth factor binding protein 5	0.391 ± 0.31	0.942 ± 0.15	0.242 ± 0.32	0.913 ± 0.42	0.651 ± 0.40
Mir136	MicroRNA 136	0.403 ± 0.49	1.306 ± 0.78	0.203 ± 0.62	0.313 ± 0.50	0.448 ± 0.47
Gm23508	Predicted gene 23508	0.408 ± 0.29	1.302 ± 0.20	0.229 ± 0.26	0.338 ± 0.53	0.404 ± 0.45
Rassf4	Ras association (RalGDS/AF-6) domain family member 4	0.419 ± 0.24	0.954 ± 0.15	1.251 ± 0.30	1.532 ± 0.35	1.654 ± 0.34
Clmn	Calmin	0.420 ± 0.42	1.246 ± 0.41	0.572 ± 0.17	0.536 ± 0.26	0.581 ± 0.22
Cyb5r2	Cytochrome b5 reductase 2	0.421 ± 0.43	0.715 ± 0.38	0.285 ± 0.17	0.312 ± 0.32	0.456 ± 0.27
Mir154	MicroRNA 154	0.422 ± 0.48	1.279 ± 0.48	0.372 ± 0.32	0.275 ± 0.31	0.384 ± 0.65
Gm22962	Predicted gene 22962	0.424 ± 0.13	1.134 ± 0.30	0.364 ± 0.20	0.435 ± 0.28	0.46 ± 0.22
Mir493	MicroRNA 493	0.425 ± 0.40	1.107 ± 0.44	0.324 ± 0.56	0.331 ± 0.37	0.338 ± 0.51
Mir543	MicroRNA 543	0.428 ± 0.76	1.359 ± 0.44	0.543 ± 0.29	0.404 ± 0.36	0.432 ± 0.51
Mir1906-1	MicroRNA 1906-1	0.429 ± 0.35	1.146 ± 0.71	0.369 ± 0.52	0.338 ± 0.31	0.433 ± 0.39
Mir485	MicroRNA 485	0.433 ± 0.51	1.466 ± 0.21	0.385 ± 0.49	0.399 ± 0.35	0.438 ± 0.27
Mir377	MicroRNA 377	0.435 ± 0.38	1.138 ± 0.31	0.362 ± 0.38	0.356 ± 0.35	0.371 ± 0.44
Cmtm5	CKLF-like MARVEL transmembrane domain 5	0.440 ± 0.33	1.103 ± 0.33	0.221 ± 0.28	0.258 ± 0.51	0.464 ± 0.36
Mir379	MicroRNA 379	0.443 ± 0.33	1.188 ± 0.25	0.420 ± 0.26	0.450 ± 0.26	0.487 ± 0.27
Mir1224	MicroRNA 1224	0.443 ± 0.34	1.167 ± 0.36	0.398 ± 0.46	0.641 ± 0.64	0.688 ± 0.54
Gm23736	Predicted gene 23736	0.444 ± 0.25	1.094 ± 0.21	0.211 ± 0.41	0.351 ± 1.02	0.519 ± 1.20
Fibin	Fin bud initiation factor homolog	0.447 ± 0.42	1.155 ± 0.29	0.849 ± 0.05	0.564 ± 0.31	0.690 ± 0.21
Lrp4	Low density lipoprotein receptor-related protein 4	0.448 ± 0.40	0.985 ± 0.27	0.327 ± 0.22	0.761 ± 0.29	1.106 ± 0.25
Gm6260	Predicted gene 6260	0.452 ± 0.45	0.949 ± 0.40	0.222 ± 0.44	0.527 ± 0.22	0.633 ± 0.23
Igdcc4	Immunoglobulin superfamily, DCC subclass, member 4	0.452 ± 0.26	1.171 ± 0.29	0.663 ± 0.25	0.530 ± 0.20	0.678 ± 0.15

Dmrta1	Doublesex and mab-3 related TF like family A1	0.460 ± 0.28	0.883 ± 0.47	0.326 ± 0.20	0.366 ± 0.35	0.457 ± 0.32
Pde7b	Phosphodiesterase 7B	0.460 ± 0.33	0.720 ± 0.24	0.408 ± 0.28	0.384 ± 0.19	0.488 ± 0.15
Mir337	MicroRNA 337	0.469 ± 0.47	1.306 ± 0.34	0.450 ± 0.58	0.393 ± 0.36	0.374 ± 0.54
Mir432	MicroRNA 432	0.470 ± 0.23	1.106 ± 0.43	0.279 ± 0.28	0.430 ± 0.53	0.538 ± 0.39
Gm22205	Predicted gene 22205	0.475 ± 0.26	1.587 ± 0.24	0.246 ± 0.40	0.306 ± 0.28	0.349 ± 0.28
Frzb	Frizzled-related protein	0.477 ± 0.61	1.259 ± 0.51	0.262 ± 0.27	0.414 ± 0.29	0.557 ± 0.23
Gjc3	Gap junction protein, gamma 3	0.479 ± 0.65	0.838 ± 0.52	0.307 ± 0.40	0.525 ± 0.46	0.666 ± 0.60
Boc	Biregional cell adhesion molecule-related by Cdon	0.480 ± 0.28	1.108 ± 0.09	0.711 ± 0.15	0.495 ± 0.24	0.542 ± 0.24
Slc17a9	Solute carrier family 17, member 9	0.490 ± 0.26	0.985 ± 0.20	0.396 ± 0.16	0.688 ± 0.17	0.680 ± 0.22
Mir667	MicroRNA 667	0.490 ± 0.33	0.973 ± 0.25	0.486 ± 0.22	0.397 ± 0.66	0.413 ± 0.59
Gm25854	Predicted gene 25854	0.493 ± 0.34	1.312 ± 0.26	0.346 ± 0.41	0.422 ± 0.40	0.448 ± 0.34
Apela	Apelin receptor early endogenous ligand	0.496 ± 0.46	0.816 ± 0.51	0.435 ± 0.24	0.560 ± 0.63	0.384 ± 0.48
Mir300	MicroRNA 300	0.497 ± 0.27	1.285 ± 0.45	0.349 ± 0.66	0.295 ± 0.71	0.324 ± 0.59
Smoc2	SPARC related modular calcium binding 2	0.497 ± 0.23	1.274 ± 0.31	0.258 ± 0.31	0.321 ± 0.32	0.480 ± 0.27
Mir382	MicroRNA 382	0.498 ± 0.29	1.619 ± 0.29	0.363 ± 0.50	0.361 ± 0.32	0.360 ± 0.40
Gm23787	Predicted gene 23787	0.501 ± 0.35	1.341 ± 0.31	0.341 ± 0.34	0.474 ± 0.37	0.599 ± 0.50
Ppp1r3c	Protein phosphatase 1, regulatory subunit 3C	0.502 ± 0.52	1.113 ± 0.37	1.171 ± 0.21	1.221 ± 0.25	0.715 ± 0.30
Mir380	MicroRNA 380	0.503 ± 0.54	1.306 ± 0.40	0.329 ± 0.43	0.393 ± 0.42	0.365 ± 0.70
Gm24564	Predicted gene 24564	0.505 ± 0.45	1.604 ± 0.18	0.257 ± 0.18	0.280 ± 0.21	0.312 ± 0.22
Cth	Cystathionase (cystathionine gamma-lyase)	0.505 ± 0.44	1.024 ± 0.30	0.540 ± 0.19	0.864 ± 0.13	0.923 ± 0.11
Osgin2	Oxidative stress induced growth inhibitor member 2	0.506 ± 0.33	1.097 ± 0.18	0.254 ± 0.30	0.605 ± 0.31	0.698 ± 0.28
Fxyd6	FXYD domain-containing ion transport regulator 6	0.506 ± 0.47	1.044 ± 0.29	0.136 ± 0.39	0.382 ± 0.29	0.424 ± 0.32
Acot2	Acyl-CoA thioesterase 2	0.508 ± 0.35	0.841 ± 0.28	0.524 ± 0.25	0.658 ± 0.23	0.813 ± 0.20
Mir3070b	MicroRNA 3070b	0.509 ± 0.38	1.167 ± 0.42	0.472 ± 0.46	0.445 ± 0.27	0.458 ± 0.47
Mir679	MicroRNA 679	0.510 ± 0.32	1.104 ± 0.31	0.539 ± 0.28	0.483 ± 0.31	0.394 ± 0.37
Sorbs2	Sorbin and SH3 domain containing 2	0.510 ± 0.11	1.154 ± 0.19	0.974 ± 0.40	0.764 ± 0.23	0.720 ± 0.19
Gm24679	Predicted gene 24679	0.514 ± 0.56	0.607 ± 0.63	0.902 ± 0.16	0.949 ± 0.41	1.124 ± 0.38
Cpa4	Carboxypeptidase A4	0.514 ± 0.46	0.882 ± 0.31	1.176 ± 0.31	1.332 ± 0.37	1.209 ± 0.23
Pdgfrl	Platelet-derived growth factor receptor-like	0.515 ± 0.21	1.118 ± 0.21	1.129 ± 0.15	1.176 ± 0.13	1.103 ± 0.08
Rab2b	RAB2B, member RAS oncogene family	0.515 ± 0.09	0.943 ± 0.19	1.109 ± 0.18	0.935 ± 0.17	0.985 ± 0.16
Car3	Carbonic anhydrase 3	0.517 ± 0.22	0.803 ± 0.70	0.516 ± 0.48	0.384 ± 0.47	0.370 ± 0.60
Meg3	Maternally expressed 3	0.518 ± 0.20	1.092 ± 0.19	0.471 ± 0.17	0.445 ± 0.24	0.501 ± 0.37
Mir1197	MicroRNA 1197	0.522 ± 0.11	1.391 ± 0.10	0.360 ± 0.57	0.368 ± 0.33	0.381 ± 0.52

Zfp185	Zinc finger protein 185	0.522 ± 0.26	1.110 ± 0.18	0.608 ± 0.41	0.584 ± 0.29	0.834 ± 0.21
Cthrc1	Collagen triple helix repeat containing 1	0.523 ± 0.18	0.948 ± 0.29	0.420 ± 0.14	0.475 ± 0.27	0.605 ± 0.21
Gm25856	Predicted gene 25856	0.523 ± 0.38	1.494 ± 0.27	0.328 ± 0.50	0.324 ± 0.32	0.383 ± 0.38
Mir453	MicroRNA 453	0.527 ± 0.33	1.156 ± 0.24	0.362 ± 0.34	0.427 ± 0.17	0.372 ± 0.32
Mir322	MicroRNA 322	0.528 ± 0.30	1.228 ± 0.24	0.971 ± 0.30	0.934 ± 0.22	1.001 ± 0.20
Svep1	Sushi, von Willebrand factor type A	0.528 ± 0.31	1.247 ± 0.26	2.385 ± 0.16	0.807 ± 0.24	0.945 ± 0.18
Cpxm2	carboxypeptidase X 2 (M14 family)	0.529 ± 0.41	1.173 ± 0.20	0.961 ± 0.14	1.053 ± 0.14	1.345 ± 0.08
Gm23600	Predicted gene 23600	0.529 ± 0.47	1.574 ± 0.28	0.328 ± 0.21	0.341 ± 0.26	0.375 ± 0.24
Dkk3	Dickkopf homolog 3	0.531 ± 0.41	0.794 ± 0.29	1.861 ± 0.18	1.031 ± 0.42	1.120 ± 0.47
AF357425	SnoRNA AF357425	0.532 ± 0.26	1.139 ± 0.15	0.394 ± 0.23	0.473 ± 0.13	0.545 ± 0.22
Nynrin	NYN domain and retroviral integrase containing	0.533 ± 0.29	1.027 ± 0.30	0.692 ± 0.18	0.503 ± 0.26	0.616 ± 0.20
Pck2	Phosphoenolpyruvate carboxykinase 2	0.533 ± 0.30	0.954 ± 0.21	0.398 ± 0.20	0.710 ± 0.16	0.761 ± 0.11
Penk	Preproenkephalin	0.534 ± 0.34	1.088 ± 0.19	1.502 ± 0.17	0.930 ± 0.15	0.922 ± 0.17
Lrrc61	Leucine rich repeat containing 61	0.537 ± 0.21	0.676 ± 0.25	0.708 ± 0.14	0.747 ± 0.18	0.880 ± 0.15
Mir376b	MicroRNA 376b	0.537 ± 0.51	1.377 ± 0.41	0.646 ± 0.21	0.295 ± 0.79	0.332 ± 0.82
Mir323	MicroRNA 323	0.537 ± 0.35	1.054 ± 0.37	0.370 ± 0.05	0.357 ± 0.67	0.362 ± 0.52
Magel2	Melanoma antigen, family L, 2	0.538 ± 0.47	1.003 ± 0.50	0.231 ± 0.30	0.410 ± 0.19	0.594 ± 0.32
Rian	RNA imprinted and accumulated in nucleus	0.538 ± 0.37	1.309 ± 0.18	0.346 ± 0.10	0.420 ± 0.26	0.489 ± 0.33
Srebf1	Sterol regulatory element binding transcription factor 1	0.539 ± 0.28	1.095 ± 0.26	0.530 ± 0.20	0.835 ± 0.14	0.852 ± 0.12
Snhg18	Small nucleolar RNA host gene 18	0.540 ± 0.30	1.109 ± 0.17	0.910 ± 0.21	0.751 ± 0.16	0.759 ± 0.16
Gm23347	Predicted gene 23347	0.542 ± 0.19	1.288 ± 0.45	0.374 ± 0.67	0.422 ± 0.30	0.427 ± 0.26
Aldh1l2	Aldehyde dehydrogenase 1 family, member L2	0.544 ± 0.30	0.994 ± 0.09	0.432 ± 0.15	0.675 ± 0.22	0.702 ± 0.19
Gm22659	Predicted gene 22659	0.544 ± 0.31	0.807 ± 0.50	1.121 ± 0.55	0.886 ± 0.30	0.935 ± 0.33
Col10a1	Collagen, type X, alpha 1	0.545 ± 0.70	0.854 ± 0.50	0.174 ± 0.20	0.416 ± 0.34	0.399 ± 0.39
AF357426	SnoRNA AF357426	0.546 ± 0.24	1.173 ± 0.21	0.485 ± 0.44	0.440 ± 0.37	0.557 ± 0.55
Sh3rf2	SH3 domain containing ring finger 2	0.546 ± 0.14	0.935 ± 0.42	0.709 ± 0.15	0.657 ± 0.22	0.818 ± 0.14
Gm24899	Predicted gene 24899	0.546 ± 0.18	1.473 ± 0.23	0.331 ± 0.21	0.427 ± 0.31	0.468 ± 0.26
Trp53inp2	Transformation related protein 53	0.547 ± 0.16	0.704 ± 0.13	1.432 ± 0.20	1.246 ± 0.13	1.276 ± 0.16
Fbxw18	F-box and WD-40 domain protein 18	0.547 ± 0.27	1.165 ± 0.24	0.328 ± 0.46	0.574 ± 0.36	0.707 ± 0.44
Omd	Osteomodulin	0.548 ± 0.24	1.072 ± 0.23	0.353 ± 0.30	0.358 ± 0.32	0.499 ± 0.29
Vmn2r5	Vomeronasal 2, receptor 5	0.548 ± 0.28	0.753 ± 0.27	0.981 ± 0.41	1.014 ± 0.42	1.395 ± 0.58
Clcn5	Chloride channel 5	0.550 ± 0.18	0.934 ± 0.18	0.587 ± 0.09	0.648 ± 0.09	0.723 ± 0.06
Rps6ka2	Ribosomal protein S6 kinase, polypeptide 2	0.550 ± 0.21	0.964 ± 0.25	0.382 ± 0.19	0.441 ± 0.34	0.650 ± 0.25

Slc16a14	Solute carrier family 16, member 14	0.550 ± 0.59	0.762 ± 0.47	0.257 ± 0.35	0.196 ± 0.45	0.289 ± 0.32
Ogn	Osteoglycin	0.551 ± 0.15	1.162 ± 0.13	0.829 ± 0.11	0.466 ± 0.33	0.663 ± 0.11
Gm13293	Predicted gene 13293	0.551 ± 0.50	1.307 ± 0.32	2.525 ± 0.24	4.398 ± 0.24	2.987 ± 0.22
Trib3	Tribbles homolog 3 (Drosophila)	0.552 ± 0.34	0.969 ± 0.25	0.546 ± 0.16	0.666 ± 0.24	0.773 ± 0.19
Cdkl5	Cyclin-dependent kinase-like 5	0.554 ± 0.11	0.932 ± 0.13	0.704 ± 0.24	0.549 ± 0.32	0.664 ± 0.26
Gm25394	Predicted gene 25394	0.556 ± 0.12	0.632 ± 0.14	0.902 ± 0.22	1.002 ± 1.94	1.010 ± 1.99
Ldlrad3	LDL receptor class A domain containing 3	0.556 ± 0.15	1.005 ± 0.12	0.364 ± 0.12	0.517 ± 0.11	0.634 ± 0.13
Aldh6a1	Aldehyde dehydrogenase family 6, subfamily A1	0.558 ± 0.29	1.214 ± 0.23	1.279 ± 0.23	0.879 ± 0.22	1.037 ± 0.17
AF357428	SnoRNA AF357428	0.559 ± 0.37	1.092 ± 0.18	0.480 ± 0.46	0.460 ± 0.60	0.549 ± 0.42
Pygo1	Pygopus 1	0.562 ± 0.19	1.195 ± 0.21	0.872 ± 0.24	0.602 ± 0.24	0.783 ± 0.23
Fam13c	Family with sequence similarity 13, member C	0.562 ± 0.24	1.000 ± 0.09	0.795 ± 0.14	0.722 ± 0.25	0.924 ± 0.16
Srpx	Sushi-repeat-containing protein	0.563 ± 0.43	1.256 ± 0.29	0.451 ± 0.32	0.423 ± 0.31	0.548 ± 0.27
Cybrd1	Cytochrome b reductase 1	0.564 ± 0.54	1.191 ± 0.29	0.317 ± 0.26	0.386 ± 0.35	0.524 ± 0.32
Creb3l1	cAMP responsive element binding protein 3-like 1	0.564 ± 0.26	0.683 ± 0.25	1.315 ± 0.04	1.150 ± 0.13	1.090 ± 0.13
Sec16b	SEC16 homolog B (S. cerevisiae)	0.565 ± 0.26	1.019 ± 0.21	0.446 ± 0.12	0.751 ± 0.25	0.746 ± 0.29
Smarca1	SWI/SNF related, matrix associated, member 1	0.565 ± 0.22	1.287 ± 0.24	0.639 ± 0.18	0.583 ± 0.24	0.623 ± 0.20
Smpd3	Sphingomyelin phosphodiesterase 3, neutral	0.566 ± 0.57	1.587 ± 0.61	0.163 ± 0.17	0.210 ± 0.37	0.304 ± 0.36
Ap3s2	Adaptor-related protein complex 3, sigma 2 subunit	0.568 ± 0.22	0.822 ± 0.22	0.985 ± 0.14	0.842 ± 0.16	0.856 ± 0.20
Hacd1	3-hydroxyacyl-CoA dehydratase 1	0.568 ± 0.15	0.975 ± 0.10	1.197 ± 0.28	0.856 ± 0.31	0.902 ± 0.33
Lctl	Lactase-like	0.571 ± 0.36	1.006 ± 0.20	0.519 ± 0.24	0.811 ± 0.32	0.981 ± 0.28
Arsi	Arylsulfatase	0.571 ± 0.10	0.814 ± 0.32	0.479 ± 0.25	0.512 ± 0.30	0.538 ± 0.35
Mir1193	MicroRNA 1193	0.571 ± 0.35	1.018 ± 0.44	0.467 ± 0.31	0.443 ± 0.36	0.463 ± 0.41
Cdon	Cell adhesion molecule-related by oncogenes	0.573 ± 0.17	1.145 ± 0.12	1.412 ± 0.11	0.836 ± 0.12	0.846 ± 0.12
Atf5	Activating transcription factor 5	0.575 ± 0.19	0.892 ± 0.17	0.794 ± 0.12	0.805 ± 0.18	0.893 ± 0.21
Camk4	Calcium/calmodulin-dependent protein kinase IV	0.576 ± 0.25	0.863 ± 0.24	0.606 ± 0.17	0.517 ± 0.24	0.645 ± 0.19
Ptgis	Prostaglandin I2 synthase	0.576 ± 0.27	1.041 ± 0.28	0.641 ± 0.14	0.733 ± 0.19	0.589 ± 0.18
Slc16a2	Solute carrier family 16, member 2	0.578 ± 0.33	1.181 ± 0.31	2.459 ± 0.08	2.224 ± 0.12	1.987 ± 0.18
Slc13a5	Solute carrier family 13, member 5	0.580 ± 0.94	1.568 ± 0.83	0.468 ± 0.49	0.985 ± 0.46	1.109 ± 0.40
Mir411	MicroRNA 411	0.581 ± 0.28	1.443 ± 0.36	0.613 ± 0.51	0.560 ± 0.34	0.587 ± 0.40
Sorbs2os	Sorbin and SH3 domain containing 2, opposite strand	0.581 ± 0.09	1.284 ± 0.19	1.100 ± 0.30	0.908 ± 0.29	0.944 ± 0.22
Fndc1	Fibronectin type III domain containing 1	0.582 ± 0.46	0.980 ± 0.33	2.794 ± 0.20	1.693 ± 0.51	1.004 ± 0.50
Mir341	MicroRNA 341	0.582 ± 0.34	1.522 ± 0.41	0.496 ± 0.22	0.545 ± 0.17	0.583 ± 0.23
6430411K18Rik	RIKEN cDNA 6430411K18 gene	0.582 ± 0.32	1.313 ± 0.16	0.527 ± 0.24	0.458 ± 0.40	0.455 ± 0.32

Gm24598	Predicted gene 24598	0.582 ± 0.36	1.538 ± 0.29	0.293 ± 0.76	0.373 ± 0.37	0.436 ± 0.46
Snord123	Small nucleolar RNA, C/D box 123	0.584 ± 0.18	0.960 ± 0.16	0.919 ± 0.11	0.924 ± 0.85	0.955 ± 0.81
Fry	Furry homolog (Drosophila)	0.585 ± 0.35	1.429 ± 0.20	0.421 ± 0.64	0.564 ± 0.46	0.571 ± 0.30
Dhrs3	Dehydrogenase/reductase (SDR family) member 3	0.585 ± 0.36	1.077 ± 0.11	1.520 ± 0.10	0.998 ± 0.12	0.907 ± 0.16
Gpc4	Glypican 4	0.585 ± 0.15	1.008 ± 0.25	0.787 ± 0.13	0.596 ± 0.18	0.666 ± 0.10
Phyh	Phytanoyl-CoA hydroxylase	0.586 ± 0.25	1.254 ± 0.14	0.573 ± 0.20	0.993 ± 0.16	0.963 ± 0.20
Scin	Scinderin	0.586 ± 0.39	1.103 ± 0.35	0.347 ± 0.12	0.711 ± 0.20	0.719 ± 0.28
Paqr3	Progestin and adipoQ receptor family member III	0.587 ± 0.56	1.095 ± 0.40	0.432 ± 0.18	0.557 ± 0.26	0.703 ± 0.16
Gm25518	Predicted gene 25518	0.587 ± 0.34	0.866 ± 0.43	1.009 ± 0.97	0.954 ± 0.59	1.277 ± 0.83
Tmem161a	Transmembrane protein 161A	0.587 ± 0.15	1.118 ± 0.17	0.870 ± 0.09	0.671 ± 0.13	0.799 ± 0.18
Mir541	MicroRNA 541	0.587 ± 0.33	1.073 ± 0.23	0.386 ± 0.42	0.490 ± 0.26	0.540 ± 0.31
Dact1	Dapper homolog 1, antagonist of beta-catenin (xenopus)	0.587 ± 0.36	0.908 ± 0.26	0.240 ± 0.34	0.394 ± 0.17	0.513 ± 0.23
Fads1	Fatty acid desaturase 1	0.589 ± 0.17	1.045 ± 0.15	0.595 ± 0.19	0.665 ± 0.13	0.771 ± 0.12
Panx3	Pannexin 3	0.589 ± 0.41	0.878 ± 0.38	0.591 ± 0.15	0.488 ± 0.42	0.585 ± 0.43
Fcgrt	Fc receptor, IgG, alpha chain transporter	0.591 ± 0.30	1.257 ± 0.27	1.667 ± 0.12	1.294 ± 0.25	1.317 ± 0.21
Plcd1	Phospholipase C, delta 1	0.591 ± 0.21	1.063 ± 0.17	0.759 ± 0.09	0.761 ± 0.10	0.813 ± 0.09
Slc35f1	Solute carrier family 35, member F1	0.592 ± 0.37	0.863 ± 0.43	0.286 ± 0.18	0.15 ± 0.23	0.203 ± 0.44
AI464131	Expressed sequence AI464131	0.592 ± 0.17	0.967 ± 0.13	0.855 ± 0.22	1.011 ± 0.16	1.026 ± 0.11
Saa3	Serum amyloid A3	0.593 ± 2.19	2.642 ± 1.59	41.471 ± 0.70	19.970 ± 0.66	19.198 ± 0.66
Copz2	Coatomer protein complex, subunit zeta 2	0.593 ± 0.15	1.019 ± 0.08	0.725 ± 0.13	0.719 ± 0.15	0.746 ± 0.15
Sh3tc2	SH3 domain and tetratricopeptide repeats 2	0.593 ± 0.34	1.049 ± 0.25	0.361 ± 0.22	0.503 ± 0.34	0.644 ± 0.24
Gm5947	Predicted pseudogene 5947	0.593 ± 0.25	0.738 ± 0.22	0.883 ± 0.30	0.966 ± 0.65	1.056 ± 0.57
Olfr1265	Olfactory receptor 1265	0.594 ± 0.29	1.045 ± 0.37	0.946 ± 0.73	0.834 ± 0.39	1.097 ± 0.31
Fkbp7	FK506 binding protein 7	0.594 ± 0.17	1.030 ± 0.15	1.024 ± 0.22	1.001 ± 0.13	0.878 ± 0.16
Mir431	MicroRNA 431	0.594 ± 0.27	1.180 ± 0.17	0.542 ± 0.17	0.555 ± 0.21	0.587 ± 0.28
Tubb2a	Tubulin, beta 2A class IIA	0.595 ± 0.23	1.038 ± 0.21	0.351 ± 0.12	0.537 ± 0.19	0.549 ± 0.29
Tbx5	T-box 5	0.595 ± 0.27	0.983 ± 0.26	0.809 ± 0.12	0.805 ± 0.38	0.781 ± 0.29
Col1a1	Collagen, type I, alpha 1	0.595 ± 0.20	0.949 ± 0.17	0.984 ± 0.16	0.937 ± 0.31	0.961 ± 0.34
Mir30e	MicroRNA 30e	0.596 ± 0.54	0.626 ± 0.41	1.078 ± 0.44	1.207 ± 0.73	1.103 ± 0.91
Itpkb	Inositol 1,4,5-trisphosphate 3-kinase B	0.596 ± 0.31	1.140 ± 0.32	0.637 ± 0.16	0.790 ± 0.14	0.787 ± 0.15
Fam213b	Family with sequence similarity 213, member B	0.596 ± 0.30	1.034 ± 0.30	0.890 ± 0.07	1.385 ± 0.39	1.219 ± 0.26
Rhox3f	Reproductive homeobox 3F	0.596 ± 0.37	0.846 ± 0.76	0.701 ± 0.48	0.846 ± 0.97	0.855 ± 0.92
Clip3	CAP-GLY domain containing linker protein 3	0.597 ± 0.21	0.877 ± 0.28	1.281 ± 0.09	0.890 ± 0.16	0.865 ± 0.22

Supplementary Table 4. List of up-regulated genes (> 1.5-fold) following knock-down of Regnase-1 via Ad-shRegnase-1A infection in chondrocytes.

Gene Symbol	Definition	Fold change				
		Ad-shRegnase-1	Ad-Regnase-1	IL-1β	Ad-HIF-2α	Ad-ZIP8
Cxcl5	Chemokine (C-X-C motif) ligand 5	3.482 ± 0.99	1.709 ± 0.70	93.775 ± 0.20	41.737 ± 0.30	43.135 ± 0.39
Saa3	Serum amyloid A 3	2.642 ± 1.59	0.593 ± 2.19	41.471 ± 0.70	19.97 ± 0.66	19.198 ± 0.66
Steap4	STEAP family member 4	2.579 ± 0.87	1.143 ± 0.62	59.772 ± 0.56	21.25 ± 0.65	21.596 ± 0.65
Gm25357	Predicted gene, 25357	2.345 ± 1.57	1.331 ± 1.14	0.956 ± 0.57	0.525 ± 1.07	0.631 ± 1.03
Mir181a-1	MicroRNA 181a-1	2.195 ± 0.72	1.155 ± 0.32	2.189 ± 0.61	0.975 ± 0.81	1.207 ± 0.90
Vmn1r127	Vomeronasal 1 receptor 127	2.152 ± 0.30	1.606 ± 0.27	0.994 ± 0.67	1.418 ± 1.00	0.916 ± 1.09
Angptl4	Angiopoietin-like 4	1.993 ± 0.60	1.218 ± 0.25	6.622 ± 0.33	7.342 ± 0.65	5.672 ± 0.61
Mmp3	Matrix metallopeptidase 3	1.847 ± 0.71	2.374 ± 0.37	157.581 ± 0.19	116.517 ± 0.58	63.661 ± 0.68
Gm23474	Predicted gene 23474	1.828 ± 0.83	0.780 ± 0.50	0.429 ± 1.08	0.363 ± 1.03	0.423 ± 1.13
Gm22740	Predicted gene 22740	1.814 ± 0.37	0.916 ± 0.66	0.632 ± 0.43	0.847 ± 0.77	0.877 ± 0.65
Gm26827	Predicted gene 26827	1.767 ± 0.34	0.838 ± 0.28	0.881 ± 0.19	1.543 ± 0.17	1.406 ± 0.21
Zscan4b	Zinc finger and SCAN domain containing 4B	1.758 ± 0.43	1.208 ± 0.34	1.237 ± 0.50	0.890 ± 0.38	0.939 ± 0.50
Mir335	MicroRNA 335	1.757 ± 0.30	1.064 ± 0.34	0.608 ± 1.17	1.168 ± 0.50	0.964 ± 0.45
Mir743	MicroRNA 743	1.709 ± 0.42	1.595 ± 0.63	1.574 ± 0.61	1.055 ± 0.51	1.167 ± 0.54
Gm4825	Predicted pseudogene 4825	1.704 ± 0.50	1.213 ± 0.46	1.215 ± 0.62	0.798 ± 0.40	0.637 ± 0.28
Gm26151	Predicted gene 26151	1.698 ± 0.48	1.242 ± 0.32	1.568 ± 0.60	1.194 ± 0.49	0.668 ± 0.37
Hp	Haptoglobin	1.696 ± 0.47	1.576 ± 0.41	67.397 ± 0.30	62.303 ± 0.30	42.068 ± 0.30
Gm22019	Predicted gene 22019	1.690 ± 0.61	1.214 ± 0.68	1.007 ± 0.39	0.806 ± 0.85	0.760 ± 0.83
Prg4	Proteoglycan 4	1.678 ± 0.95	2.019 ± 0.72	7.197 ± 0.16	8.533 ± 0.48	3.577 ± 0.57
Gm25724	Predicted gene, 25724	1.636 ± 0.35	1.309 ± 0.28	0.934 ± 0.43	0.708 ± 0.56	0.522 ± 0.34
mt-Ts2	Mitochondrially encoded tRNA serine 2	1.625 ± 0.66	2.011 ± 0.79	0.825 ± 0.96	0.827 ± 0.43	0.905 ± 0.55
Mir382	MicroRNA 382	1.619 ± 0.29	0.498 ± 0.29	0.363 ± 0.50	0.361 ± 0.32	0.360 ± 0.40
Gm24960	Predicted gene 24960	1.614 ± 0.13	1.323 ± 0.51	0.796 ± 0.55	1.243 ± 0.77	1.637 ± 0.80
Gm23297	Predicted gene 23297	1.612 ± 0.24	1.393 ± 0.40	1.207 ± 0.14	1.003 ± 0.68	0.821 ± 0.82
Mir1955	MicroRNA 1955	1.611 ± 0.44	1.122 ± 0.27	1.546 ± 0.67	1.128 ± 0.25	1.108 ± 0.25
Gm24564	Predicted gene, 24564	1.604 ± 0.18	0.505 ± 0.45	0.257 ± 0.18	0.28 ± 0.21	0.312 ± 0.22
Chil1	Chitinase-like 1	1.599 ± 1.09	0.722 ± 1.61	15.791 ± 0.37	9.608 ± 0.43	8.597 ± 0.43
Mir186	MicroRNA 186	1.595 ± 0.55	1.511 ± 0.47	1.553 ± 0.32	0.653 ± 0.34	0.679 ± 0.76
Mirlet7c-1	MicroRNA let7c-1	1.594 ± 0.40	0.772 ± 0.27	1.111 ± 0.68	1.025 ± 0.47	1.058 ± 0.17
Smpd3	Sphingomyelin phosphodiesterase 3	1.587 ± 0.61	0.566 ± 0.57	0.163 ± 0.17	0.210 ± 0.37	0.304 ± 0.36
Gm22205	Predicted gene 22205	1.587 ± 0.24	0.475 ± 0.26	0.246 ± 0.40	0.306 ± 0.28	0.349 ± 0.28
Vnn1	Vanin 1	1.586 ± 0.49	0.994 ± 0.49	61.952 ± 0.25	13.662 ± 0.57	14.634 ± 0.56
Gm22263	Predicted gene 22263	1.584 ± 0.26	0.97 ± 0.24	0.902 ± 0.85	1.245 ± 0.81	0.838 ± 0.82
Trbj1-7	T cell receptor beta joining 1-7	1.583 ± 0.73	1.043 ± 0.67	1.144 ± 0.28	0.895 ± 0.37	0.743 ± 0.46

Gm10944	Predicted gene 10944	1.582 ± 0.74	1.259 ± 1.05	1.287 ± 0.68	0.738 ± 0.96	0.726 ± 1.17
Gm5174	Predicted gene 5174	1.580 ± 0.41	1.171 ± 0.15	1.312 ± 0.25	1.074 ± 0.32	1.103 ± 0.26
Sema3d	Sema domain, Ig domain, short basic domain	1.578 ± 0.34	1.018 ± 0.33	0.321 ± 0.47	0.786 ± 0.36	0.645 ± 0.28
Gm23600	Predicted gene 23600	1.574 ± 0.28	0.529 ± 0.47	0.328 ± 0.21	0.341 ± 0.26	0.375 ± 0.24
Slc13a5	Solute carrier family 13, member 5	1.568 ± 0.83	0.580 ± 0.94	0.468 ± 0.49	0.985 ± 0.46	1.109 ± 0.4
Mir466e	MicroRNA 466e	1.563 ± 0.47	1.111 ± 0.43	1.457 ± 0.64	1.166 ± 0.57	0.680 ± 0.60
Ddx3y	DEAD (Asp-Glu-Ala-Asp) box polypeptide 3, Y-linked	1.563 ± 0.98	1.413 ± 0.98	0.870 ± 0.54	0.921 ± 0.31	0.869 ± 0.29
Gm23199	Predicted gene 23199	1.560 ± 0.36	1.207 ± 0.37	0.603 ± 0.53	0.892 ± 0.39	1.043 ± 0.13
Klk1b27	Kallikrein 1-related peptidase b27	1.558 ± 0.37	1.360 ± 0.54	1.086 ± 0.35	0.988 ± 0.40	1.092 ± 0.67
Uty	Ubiquitously transcribed tetratricopeptide repeat gene	1.556 ± 0.81	1.386 ± 0.81	1.393 ± 0.55	1.056 ± 0.39	0.948 ± 0.38
Stfa3	Stefin A3	1.551 ± 0.33	1.065 ± 0.25	0.674 ± 0.27	0.892 ± 0.54	0.978 ± 0.47
Gm24599	Predicted gene 24599	1.549 ± 0.42	1.111 ± 0.32	0.917 ± 0.48	1.149 ± 0.32	1.237 ± 0.46
Gm24561	Predicted gene 24561	1.546 ± 1.13	2.500 ± 0.61	1.184 ± 1.05	1.078 ± 0.56	1.443 ± 0.59
Mir130a	MicroRNA 130a	1.541 ± 0.60	0.787 ± 0.21	1.046 ± 0.49	0.638 ± 0.59	0.933 ± 0.49
DQ267100	SnoRNA DQ267100	1.540 ± 0.20	0.619 ± 0.26	0.343 ± 0.20	0.477 ± 0.31	0.508 ± 0.23
Gm24598	Predicted gene 24598	1.538 ± 0.29	0.582 ± 0.36	0.293 ± 0.76	0.373 ± 0.37	0.436 ± 0.46
Oxct2a	3-oxoacid CoA transferase 2A	1.537 ± 0.37	1.148 ± 0.28	0.828 ± 0.33	0.974 ± 0.54	1.157 ± 0.62
Aspn	Asporin	1.537 ± 0.68	1.049 ± 0.57	0.307 ± 0.51	0.261 ± 1.30	0.252 ± 1.19
Phtf1os	Putative homeodomain transcription factor 1	1.529 ± 0.19	1.419 ± 0.13	1.121 ± 0.26	0.989 ± 0.13	0.979 ± 0.4
Mir450-1	MicroRNA 450-1	1.528 ± 0.47	1.133 ± 0.39	1.081 ± 0.49	1.04 ± 0.49	0.836 ± 0.33
Saa2	Serum amyloid A 2	1.526 ± 0.54	1.257 ± 0.39	60.528 ± 0.10	94.964 ± 0.74	55.081 ± 0.76
Mterf1a	Mitochondrial transcription termination factor 1a	1.524 ± 0.57	1.377 ± 0.31	1.605 ± 0.24	0.823 ± 0.32	0.992 ± 0.42
Eif2s3y	Eukaryotic translation initiation factor 2, subunit 3	1.524 ± 0.96	1.299 ± 0.95	0.903 ± 0.84	0.834 ± 0.26	0.919 ± 0.29
Gm3715	Predicted gene 3715	1.524 ± 0.51	1.263 ± 0.49	1.201 ± 0.24	1.146 ± 0.38	1.124 ± 0.57
Mir341	MicroRNA 341	1.522 ± 0.41	0.582 ± 0.34	0.496 ± 0.22	0.545 ± 0.17	0.583 ± 0.23
Gm24475	Predicted gene 24475	1.516 ± 0.68	1.230 ± 0.60	1.102 ± 0.37	1.103 ± 0.86	0.904 ± 0.86
Gm5316	Predicted gene 5316	1.515 ± 0.27	1.176 ± 0.40	1.041 ± 0.20	1.941 ± 0.37	1.691 ± 0.29
Gm24391	Predicted gene 24391	1.515 ± 0.27	1.150 ± 0.30	1.179 ± 0.17	1.095 ± 0.33	0.834 ± 0.63
Traj7	T cell receptor alpha joining 7	1.510 ± 0.70	1.307 ± 0.41	0.874 ± 0.53	0.806 ± 0.73	0.762 ± 0.93
Snora44	Small nucleolar RNA, H/ACA box 44	1.508 ± 0.25	1.095 ± 0.31	0.985 ± 0.10	1.990 ± 0.75	1.794 ± 0.87
Cxcl3	Chemokine (C-X-C motif) ligand 3	1.506 ± 0.97	2.377 ± 0.59	251.887 ± 0.48	80.900 ± 0.55	56.658 ± 0.71
Gm16427	Predicted gene 16427	1.501 ± 0.55	1.283 ± 0.55	1.288 ± 0.48	1.282 ± 0.51	1.081 ± 0.42
Gm22296	Predicted gene 22296	1.501 ± 0.30	1.100 ± 0.19	0.935 ± 0.13	1.114 ± 0.71	1.070 ± 0.81

Supplementary Table 5. List of up-regulated genes (> 2.0-fold) following overexpression of Regnase-1 via Ad-Regnase-1 infection in chondrocytes.

Gene symbol	Definition	Fold change				
		Ad-Regnase-1	Ad-shRegnase-1	IL-1β	Ad-HIF-2α	Ad-ZIP8
Hspa1a	Heat shock protein 1A	21.073 ± 0.39	0.830 ± 0.37	1.554 ± 0.39	2.249 ± 0.45	12.68 ± 0.58
Hspa1b	Heat shock protein 1B	11.706 ± 0.36	0.872 ± 0.32	2.001 ± 0.2	2.594 ± 0.46	9.391 ± 0.41
Gm13238	Carnitine deficiency-associated gene expressed in ventricle	7.051 ± 0.58	0.872 ± 0.22	0.822 ± 0.77	0.698 ± 0.49	0.776 ± 0.37
Gpnmb	Glycoprotein (transmembrane) nmb	5.007 ± 0.61	1.138 ± 0.40	1.740 ± 0.52	3.896 ± 0.96	3.736 ± 0.96
Kitl	Kit ligand	4.790 ± 0.40	0.653 ± 0.65	5.222 ± 0.44	2.417 ± 0.34	1.719 ± 0.41
Nov	Nephroblastoma overexpressed gene	4.757 ± 0.63	0.874 ± 0.39	2.053 ± 0.16	1.643 ± 0.39	1.659 ± 0.44
Gm3776	Predicted gene 3776	4.726 ± 0.55	1.103 ± 0.61	0.963 ± 0.25	2.255 ± 0.67	2.36 ± 0.72
Zc3h12a	Zinc finger CCCH type containing 12A	4.327 ± 0.14	0.870 ± 0.47	7.781 ± 0.14	4.744 ± 0.47	3.949 ± 0.44
Ptpn22	Protein tyrosine phosphatase, non-receptor type 22	4.302 ± 0.66	0.863 ± 0.45	1.052 ± 0.27	1.040 ± 0.25	1.087 ± 0.13
Fosl1	Fos-like antigen 1	4.296 ± 0.36	0.727 ± 0.07	8.040 ± 0.26	7.144 ± 0.53	3.244 ± 0.43
Ly6c1	Lymphocyte antigen 6 complex, locus C1	4.033 ± 0.47	1.201 ± 0.11	1.060 ± 0.28	1.343 ± 0.5	1.005 ± 0.28
Serpinb2	Serine (or cysteine) peptidase inhibitor, clade B, member 2	3.882 ± 0.57	1.121 ± 0.41	1.272 ± 0.18	1.526 ± 0.42	1.200 ± 0.26
Ifi204	Interferon activated gene 204	3.859 ± 0.28	0.827 ± 0.50	6.508 ± 0.64	8.860 ± 0.38	6.310 ± 0.49
Gm20481	Predicted gene 20481	3.782 ± 0.28	1.027 ± 0.20	1.101 ± 0.37	1.634 ± 0.49	3.131 ± 0.46
Snora17	Small nucleolar RNA, H/ACA box 17	3.508 ± 0.25	1.123 ± 0.39	0.951 ± 0.10	1.133 ± 0.51	1.057 ± 0.49
Serpinb9e	Serine (or cysteine) peptidase inhibitor, clade B, member 9e	3.476 ± 0.33	0.838 ± 0.28	1.303 ± 0.32	1.395 ± 0.71	1.161 ± 0.43
Slitrk6	SLIT and NTRK-like family, member 6	3.294 ± 0.31	0.951 ± 0.12	14.520 ± 0.16	5.640± 0.44	2.330 ± 0.48
Tnfrsf23	Tumor necrosis factor receptor superfamily, member 23	3.247 ± 0.20	0.717 ± 0.43	1.717 ± 0.13	2.319 ± 0.39	1.563 ± 0.33
U90926	cDNA sequence U90926	3.242 ± 0.45	0.942 ± 0.3	24.151 ± 0.32	6.695 ± 0.62	6.18 ± 0.59
Gm2115	Predicted gene 2115	3.220 ± 0.51	0.785 ± 0.70	4.873 ± 0.20	3.051 ± 0.41	1.932 ± 0.35
Mnda	Myeloid cell nuclear differentiation antigen	3.217 ± 0.18	0.953 ± 0.16	3.804 ± 0.31	5.703 ± 0.33	3.444 ± 0.50
Dusp6	Dual specificity phosphatase 6	3.195 ± 0.37	0.675 ± 0.42	2.666 ± 0.22	2.388 ± 0.52	2.428 ± 0.49
S100a4	S100 calcium binding protein A4	3.106 ± 0.59	0.834 ± 0.44	1.669 ± 0.21	2.489 ± 0.37	2.133 ± 0.47
Ndst3	N-deacetylase/N-sulfotransferase	3.092 ± 0.63	0.874 ± 0.31	0.948 ± 0.27	1.007 ± 0.26	1.111 ± 0.13
Lrrn3	Leucine rich repeat protein 3, neuronal	3.068 ± 0.61	0.675 ± 0.48	0.979 ± 0.30	1.700 ± 0.30	2.129 ± 0.25
Pla2g7	Phospholipase A2, group VII	3.004 ± 0.28	0.938 ± 0.67	0.570 ± 0.24	1.200 ± 0.36	0.851 ± 0.31
Cd68	CD68 antigen	2.944 ± 0.27	0.864 ± 0.52	2.327 ± 0.15	1.725 ± 0.55	1.782 ± 0.29

Gm6634	Predicted gene 6634	2.911 ± 0.48	0.917 ± 0.51	1.780 ± 0.40	1.713 ± 0.35	2.417 ± 0.33
Rtp4	Receptor transporter protein 4	2.905 ± 1.02	0.957 ± 0.28	1.194 ± 0.13	1.282 ± 0.24	1.292 ± 0.18
Tnfrsf22	Tumor necrosis factor receptor superfamily, member 22	2.888 ± 0.17	0.811 ± 0.27	1.186 ± 0.22	1.244 ± 0.32	1.478 ± 0.23
Ccnd1	Cyclin D1	2.860 ± 0.30	0.779 ± 0.38	0.610 ± 0.19	0.579 ± 0.25	0.816 ± 0.19
Tmem74	Transmembrane protein 74	2.854 ± 0.21	0.695 ± 0.22	3.009 ± 0.29	2.863 ± 0.56	1.537 ± 0.40
Hmga1-rs1	High mobility group AT-hook I, related sequence 1	2.834 ± 0.17	0.729 ± 0.19	1.274 ± 0.32	1.837 ± 0.40	1.832 ± 0.36
LOC102638521	60S ribosomal protein L23a	2.828 ± 0.26	1.070 ± 0.45	0.717 ± 0.18	0.865 ± 0.34	0.949 ± 0.39
Ly6c2	Lymphocyte antigen 6 complex, locus C2	2.801 ± 0.97	0.902 ± 0.13	0.825 ± 0.28	2.441 ± 0.43	1.221 ± 0.38
Sncg	Synuclein, gamma	2.779 ± 0.55	0.673 ± 0.23	1.759 ± 0.33	1.876 ± 0.59	1.653 ± 0.47
Hmga2-ps1	High mobility group AT-hook 2, pseudogene 1	2.775 ± 0.47	1.008 ± 0.36	1.420 ± 0.30	3.505 ± 0.57	1.332 ± 0.20
Slfn9	Schlafen 9	2.773 ± 0.34	0.725 ± 1.04	0.503 ± 0.29	3.184 ± 0.38	3.390 ± 0.36
Ptgs2	Prostaglandin-endoperoxide synthase 2	2.766 ± 0.53	0.862 ± 0.49	7.772 ± 0.16	2.793 ± 0.42	2.255 ± 0.37
Ank	Progressive ankyloses	2.750 ± 0.23	0.921 ± 0.36	0.967 ± 0.10	1.583 ± 0.32	1.381 ± 0.20
Prrg4	Proline rich Gla (G-carboxyglutamic acid) 4	2.738 ± 0.33	0.796 ± 0.58	1.368 ± 0.24	1.286 ± 0.36	0.941 ± 0.21
Adam8	A disintegrin and metallopeptidase domain 8	2.736 ± 0.26	0.714 ± 0.52	1.250 ± 0.22	1.546 ± 0.17	1.082 ± 0.35
Ddias	DNA damage-induced apoptosis suppressor	2.713 ± 0.38	0.632 ± 0.08	2.248 ± 0.33	2.074 ± 0.44	1.852 ± 0.32
Spp1	Secreted phosphoprotein 1	2.687 ± 0.36	1.031 ± 0.34	4.198 ± 0.28	4.731 ± 0.34	3.522 ± 0.37
Gm25053	Predicted gene 25053	2.678 ± 0.34	0.955 ± 0.21	0.933 ± 0.19	1.080 ± 0.64	1.173 ± 0.55
Cd34	CD34 antigen	2.667 ± 0.49	1.057 ± 0.58	0.812 ± 0.23	1.178 ± 0.54	1.608 ± 0.55
Ifi202b	Interferon activated gene 202B	2.633 ± 0.24	1.078 ± 0.35	4.270 ± 0.42	7.271 ± 0.49	4.653 ± 0.57
Layn	Layilin	2.631 ± 0.39	0.823 ± 0.24	2.497 ± 0.20	3.835 ± 0.36	2.092 ± 0.34
Eda2r	Ectodysplasin A2 receptor	2.631 ± 0.39	0.734 ± 0.30	4.225 ± 0.33	1.209 ± 0.38	1.125 ± 0.21
Mcpt8	Mast cell protease 8	2.628 ± 0.33	1.099 ± 0.23	1.203 ± 0.12	1.246 ± 0.36	1.357 ± 0.27
Ngf	Nerve growth factor	2.625 ± 0.24	0.766 ± 0.37	2.202 ± 0.28	1.530 ± 0.64	1.123 ± 0.50
Zfp52	Zinc finger protein 52	2.611 ± 0.36	0.880 ± 0.28	2.020 ± 0.50	1.684 ± 0.51	1.329 ± 0.57
Ahnak2	AHNAK nucleoprotein 2	2.602 ± 0.41	1.243 ± 0.24	2.066 ± 0.70	1.675 ± 0.60	1.528 ± 0.63
Glipr1	GLI pathogenesis-related 1	2.600 ± 0.56	0.612 ± 0.19	4.190 ± 0.30	5.148 ± 0.55	3.606 ± 0.54
Cadm1	Cell adhesion molecule 1	2.597 ± 0.44	0.891 ± 0.31	0.976 ± 0.10	0.856 ± 0.13	0.821 ± 0.17
Gm12603	Predicted gene 12603	2.594 ± 0.64	1.195 ± 0.31	1.047 ± 0.17	0.861 ± 0.28	0.873 ± 0.28
Pi15	Peptidase inhibitor 15	2.580 ± 0.32	0.922 ± 0.62	1.598 ± 0.46	0.953 ± 0.38	0.961 ± 0.28

Gm8074	Predicted gene 8074	2.571 ± 0.35	1.139 ± 0.28	0.802 ± 0.24	1.407 ± 0.54	1.445 ± 0.50
Mlkl	Mixed lineage kinase domain-like	2.538 ± 0.31	0.772 ± 0.18	1.473 ± 0.34	1.719 ± 0.47	1.414 ± 0.44
Cd44	CD44 antigen	2.530 ± 0.42	0.649 ± 0.44	2.583 ± 0.13	2.607 ± 0.18	2.334 ± 0.23
9930111J21 Rik2	RIKEN cDNA 9930111J21 gene 2	2.517 ± 1.14	0.927 ± 0.29	1.020 ± 0.29	0.936 ± 0.38	1.018 ± 0.41
Trgj4	T cell receptor gamma joining 4	2.509 ± 0.38	0.917 ± 0.31	1.125 ± 0.27	0.989 ± 0.11	1.068 ± 0.17
Ifi203	Interferon activated gene 203	2.502 ± 0.27	0.958 ± 0.41	1.147 ± 0.28	1.368 ± 0.15	1.213 ± 0.18
Gm24561	Predicted gene 24561	2.500 ± 0.61	1.546 ± 0.30	1.184 ± 1.05	1.078 ± 0.56	1.443 ± 0.59
Xdh	Xanthine dehydrogenase	2.494 ± 0.43	1.408 ± 0.21	12.478 ± 0.37	14.346 ± 0.38	10.058 ± 0.55
Gm1045	Predicted gene 1045	2.493 ± 0.26	1.185 ± 0.30	1.209 ± 0.40	1.26 ± 0.50	1.000 ± 0.42
Cdh2	Cadherin 2	2.467 ± 0.32	0.769 ± 1.13	1.375 ± 0.13	1.134 ± 0.26	1.160 ± 0.24
Rerg	RAS-like, estrogen-regulated, growth-inhibitor	2.467 ± 0.31	0.898 ± 0.72	1.283 ± 0.31	1.700 ± 0.33	1.698 ± 0.29
Nrg1	Neuregulin 1	2.440 ± 0.50	0.747 ± 0.19	4.055 ± 0.30	1.244 ± 0.18	1.659 ± 0.26
Abcb1b	ATP-binding cassette, sub-family B, member 1B	2.438 ± 0.54	0.675 ± 0.32	1.792 ± 0.44	1.478 ± 0.37	1.339 ± 0.26
Igkj1	Immunoglobulin kappa joining 1	2.421 ± 0.27	0.999 ± 0.19	1.106 ± 0.25	0.996 ± 0.52	0.906 ± 0.45
Ifit1	Interferon-induced protein with tetratricopeptide repeats 1	2.414 ± 0.49	0.796 ± 0.54	1.094 ± 0.32	1.317 ± 0.24	1.173 ± 0.25
Hmga2	High mobility group AT-hook 2	2.412 ± 0.52	0.729 ± 0.58	1.022 ± 0.18	1.727 ± 0.31	1.639 ± 0.27
Gm5424	Argininosuccinate synthase pseudogene	2.410 ± 0.27	0.732 ± 0.29	1.723 ± 0.12	1.288 ± 0.22	1.209 ± 0.41
Ppargc1a	Peroxisome proliferative activated receptor, gamma	2.406 ± 0.47	0.973 ± 0.34	0.813 ± 0.27	1.098 ± 0.40	1.294 ± 0.34
Stom	stomatin	2.404 ± 0.30	0.798 ± 0.43	2.430 ± 0.18	3.980 ± 0.35	2.138 ± 0.36
Il18rap	interleukin 18 receptor accessory protein	2.400 ± 0.39	0.923 ± 0.32	2.242 ± 0.24	1.785 ± 0.42	1.446 ± 0.26
Hspa4l	Heat shock protein 4 like	2.388 ± 0.53	0.967 ± 0.60	2.093 ± 0.37	2.417 ± 0.34	1.937 ± 0.32
Tigit	T cell immunoreceptor with Ig and ITIM domains	2.385 ± 0.40	0.911 ± 0.21	0.967 ± 0.28	0.935 ± 0.31	1.080 ± 0.29
Cd55	CD55 molecule, decay accelerating factor	2.383 ± 0.31	0.863 ± 0.35	1.068 ± 0.44	0.570 ± 0.27	0.545 ± 0.54
Slc16a10	Solute carrier family 16, member 10	2.378 ± 0.40	0.977 ± 0.29	1.087 ± 0.09	1.613 ± 0.35	1.692 ± 0.31
Cxcl3	Chemokine (C-X-C motif) ligand 3	2.377 ± 0.59	1.506 ± 0.38	251.887 ± 0.48	80.900 ± 0.55	56.658 ± 0.71
Mmp3	Matrix metallopeptidase 3	2.374 ± 0.37	1.847 ± 0.09	[illegible]57.581 ± 0.19	116.517 ± 0.58	63.661 ± 0.68
Gbp7	Guanylate binding protein 7	2.366 ± 0.81	0.887 ± 0.20	2.549 ± 0.35	2.181 ± 0.37	1.924 ± 0.38
Perp	PERP, TP53 apoptosis effector	2.334 ± 0.40	0.617 ± 0.97	1.493 ± 0.23	0.798 ± 0.32	0.667 ± 0.26
Ranbp3l	RAN binding protein 3-like	2.327 ± 0.31	1.101 ± 0.71	1.481 ± 0.13	4.460 ± 0.68	3.028 ± 0.55
Cyp1b1	Cytochrome P450, family 1, subfamily b, polypeptide 1	2.305 ± 0.29	1.149 ± 0.67	3.505 ± 0.27	2.026 ± 0.36	1.889 ± 0.47

Errfi1	ERBB receptor feedback inhibitor 1	2.303 ± 0.10	0.854 ± 0.36	3.102 ± 0.19	2.155 ± 0.26	1.512 ± 0.16
Gprc5a	G protein-coupled receptor, family C, group 5, member A	2.280 ± 0.29	0.710 ± 0.20	1.514 ± 0.37	1.501 ± 0.26	1.472 ± 0.32
Dynap	Dynactin associated protein	2.270 ± 0.59	0.948 ± 0.29	0.849 ± 0.24	0.868 ± 0.20	0.948 ± 0.16
Procr	Protein C receptor, endothelial	2.267 ± 0.25	0.996 ± 0.14	1.359 ± 0.15	1.394 ± 0.22	1.210 ± 0.23
Rgs16	Regulator of G-protein signaling 16	2.258 ± 0.25	1.047 ± 0.49	3.366 ± 0.37	1.844 ± 0.19	2.863 ± 0.29
S100a7a	S100 calcium binding protein A7A	2.246 ± 0.59	0.761 ± 0.29	1.001 ± 0.32	0.949 ± 0.15	0.993 ± 0.19
Sat1	Spermidine/spermine N1-acetyl transferase 1	2.218 ± 0.28	0.899 ± 0.16	1.401 ± 0.27	2.119 ± 0.30	1.938 ± 0.28
Pdpn	Podoplanin	2.214 ± 0.34	1.031 ± 0.17	1.521 ± 0.14	2.542 ± 0.28	1.835 ± 0.27
Serpinb8	Serine (or cysteine) peptidase inhibitor, clade B, member 8	2.205 ± 0.72	0.836 ± 0.20	0.884 ± 0.21	1.682 ± 0.16	1.573 ± 0.38
Fabp4	Fatty acid binding protein 4, adipocyte	2.197 ± 0.83	1.171 ± 0.44	11.881 ± 0.64	22.810 ± 0.91	6.871 ± 0.91
2810474O19 Rik	RIKEN cDNA 2810474O19 gene	2.197 ± 0.11	0.787 ± 0.23	0.993 ± 0.38	1.166 ± 0.17	1.218 ± 0.18
Gzme	Granzyme E	2.196 ± 1.32	0.830 ± 0.38	1.024 ± 0.14	1.205 ± 0.87	1.460 ± 0.82
Itgb7	Integrin beta 7	2.189 ± 0.19	0.963 ± 0.66	1.174 ± 0.13	1.231 ± 0.42	1.148 ± 0.15
Samd5	Sterile alpha motif domain containing 5	2.183 ± 0.27	0.587 ± 0.80	1.122 ± 0.16	1.149 ± 0.32	1.310 ± 0.25
Dsp	Desmoplakin	2.182 ± 0.63	0.646 ± 0.13	0.691 ± 0.26	1.744 ± 0.35	0.778 ± 0.24
Gcnt4	Glucosaminyl transferase 4, core 2	2.182 ± 0.35	0.694 ± 0.55	1.202 ± 0.44	1.781 ± 0.49	1.501 ± 0.32
Angptl2	Angiopoietin-like 2	2.181 ± 0.32	0.879 ± 0.2	1.163 ± 0.20	1.529 ± 0.34	0.633 ± 0.22
Gm8979	Very large inducible GTPase 1 pseudogene	2.177 ± 0.48	0.838 ± 0.17	0.869 ± 0.28	0.892 ± 0.22	0.992 ± 0.26
Adm	Adrenomedullin	2.169 ± 0.30	0.964 ± 0.6	13.538 ± 0.24	17.308 ± 0.52	4.192 ± 0.4
Kcnn4	Potassium intermediate/small conductance calcium-activated	2.159 ± 0.25	0.919 ± 0.36	0.927 ± 0.24	1.331 ± 0.20	1.030 ± 0.14
Fam111a	Family with sequence similarity 111, member A	2.158 ± 0.38	0.695 ± 0.30	0.688 ± 0.21	3.163 ± 0.35	3.350 ± 0.36
Ccl5	Chemokine (C-C motif) ligand 5	2.141 ± 0.43	0.913 ± 0.35	20.147 ± 0.68	20.685 ± 0.26	13.567 ± 0.35
Tm4sf1	Transmembrane 4 superfamily member 1	2.135 ± 0.43	0.846 ± 0.42	2.592 ± 0.14	5.148 ± 0.49	3.841 ± 0.45
P2rx7	Purinergic receptor P2X, ligand-gated ion channel, 7	2.130 ± 0.24	0.839 ± 0.12	1.849 ± 0.21	1.276 ± 0.28	1.402 ± 0.38
Met	Met proto-oncogene	2.126 ± 0.26	1.030 ± 0.49	1.105 ± 0.19	1.564 ± 0.28	1.335 ± 0.31
Gm34039	Predicted gene 34039	2.124 ± 0.32	0.906 ± 0.41	1.393 ± 0.17	1.171 ± 0.27	1.086 ± 0.22
Ifih1	Interferon induced with helicase C domain 1	2.114 ± 0.47	0.954 ± 0.46	1.637 ± 0.29	1.559 ± 0.25	1.461 ± 0.27
2010300F17 Rik	RIKEN cDNA 2010300F17 gene	2.111 ± 1.14	0.903 ± 0.22	0.543 ± 0.38	0.824 ± 0.41	0.872 ± 0.53
Dtna	Dystrobrevin alpha	2.111 ± 0.48	1.060 ± 0.21	1.718 ± 0.17	1.264 ± 0.25	1.221 ± 0.30
Dock11	Dedicator of cytokinesis 11	2.110 ± 0.27	0.796 ± 0.26	1.748 ± 0.51	1.823 ± 0.25	1.618 ± 0.27

S1pr3	Sphingosine-1-phosphate receptor 3	2.108 ± 0.23	0.973 ± 0.17	0.926 ± 0.11	0.992 ± 0.14	0.992 ± 0.11
Ccnf	Cyclin F	2.106 ± 0.27	0.780 ± 1.01	0.756 ± 0.14	1.594 ± 0.19	1.634 ± 0.21
Rrm2	Ribonucleotide reductase M2	2.104 ± 0.39	0.714 ± 0.20	0.594 ± 0.25	2.495 ± 0.33	2.678 ± 0.34
Kctd12	Potassium channel tetramerisation domain	2.099 ± 0.50	0.648 ± 0.19	3.137 ± 0.45	1.773 ± 0.23	1.982 ± 0.18
Bmpr1b	Bone morphogenetic protein receptor, type 1B	2.096 ± 0.27	1.008 ± 0.38	1.323 ± 0.29	1.581 ± 0.25	1.491 ± 0.42
Sh3kbp1	SH3-domain kinase binding protein 1	2.095 ± 0.15	0.771 ± 0.35	1.624 ± 0.11	1.461 ± 0.20	0.981 ± 0.13
Diaph3	Diaphanous related formin 3	2.079 ± 0.27	0.773 ± 0.51	0.765 ± 0.16	2.140 ± 0.33	2.171 ± 0.32
Il1rl1	Interleukin 1 receptor-like 1	2.077 ± 0.68	0.823 ± 0.60	2.574 ± 0.41	1.451 ± 0.42	1.706 ± 0.40
Itga2	Integrin alpha 2	2.074 ± 0.68	0.972 ± 0.33	0.316 ± 0.36	0.688 ± 0.14	0.623 ± 0.28
Ddit4l	DNA-damage-inducible transcript 4-like	2.069 ± 0.18	1.019 ± 0.10	3.449 ± 0.34	1.539 ± 0.33	1.410 ± 0.30
Masp1	Mannan-binding lectin serine peptidase 1	2.061 ± 0.50	0.680 ± 0.41	1.639 ± 0.26	1.277 ± 0.32	1.252 ± 0.36
Il13ra1	Interleukin 13 receptor, alpha 1	2.059 ± 0.36	1.237 ± 0.67	7.171 ± 0.29	14.384 ± 0.37	5.319 ± 0.35
Cks2	CDC28 protein kinase regulatory subunit 2	2.050 ± 0.46	0.917 ± 0.70	0.679 ± 0.47	2.040 ± 0.31	2.195 ± 0.32
Gfra1	Glial cell line derived neurotrophic factor	2.043 ± 0.23	0.864 ± 0.40	1.097 ± 0.12	1.754 ± 0.17	1.329 ± 0.30
mt-Tf	Mitochondrially encoded tRNA phenylalanine	2.033 ± 0.40	1.151 ± 0.58	2.290 ± 0.47	1.778 ± 0.15	1.314 ± 0.30
Pdgfc	Platelet-derived growth factor, C polypeptide	2.029 ± 0.26	0.936 ± 0.58	1.674 ± 0.16	1.658 ± 0.19	1.354 ± 0.18
Prg4	Proteoglycan 4	2.019 ± 0.72	1.678 ± 0.58	7.197 ± 0.16	8.533 ± 0.48	3.577 ± 0.57
Ptgs1	Prostaglandin-endoperoxide synthase 1	2.012 ± 0.23	0.927 ± 0.37	1.167 ± 0.23	1.986 ± 0.47	1.268 ± 0.36
mt-Ts2	Mitochondrially encoded tRNA serine 2	2.011 ± 0.79	1.625 ± 0.38	0.825 ± 0.96	0.827 ± 0.43	0.905 ± 0.55
Hist1h3g	Histone cluster 1, H3g	2.008 ± 0.63	0.930 ± 0.33	0.482 ± 0.54	1.309 ± 0.58	1.546 ± 0.51
Hhip	Hedgehog-interacting protein	2.006 ± 0.49	1.104 ± 0.95	0.807 ± 0.30	0.495 ± 0.42	0.539 ± 0.39
Nabp1	Nucleic acid binding protein 1	2.006 ± 0.34	0.760 ± 0.18	2.606 ± 0.21	1.707 ± 0.32	1.557 ± 0.38
Hmox1	Heme oxygenase 1	2.003 ± 0.15	1.132 ± 0.66	1.484 ± 0.11	0.908 ± 0.23	1.682 ± 0.32

Supplementary Table 6. List of down-regulated genes (< 0.7-fold) following knock-down of Regnase-1 via Ad-shRegnase-1 infection in chondrocytes.

Gene Symbol	Definition	Fold change				
		Ad-shRegnase-1	Ad-Regnase-1	IL-1β	Ad-HIF-2α	Ad-ZIP8
Cd80	CD80 antigen	0.398 ± 0.41	1.663 ± 0.39	6.498 ± 0.19	2.802 ± 0.68	2.104 ± 0.54
Hist1h2bg	Histone cluster 1, H2bg	0.464 ± 0.28	1.095 ± 0.23	0.744 ± 0.46	1.788 ± 0.58	1.740 ± 0.56
Krt19	Keratin 19	0.481 ± 0.70	1.761 ± 0.40	1.050 ± 0.41	1.633 ± 0.28	0.828 ± 0.22
Gm23470	Predicted gene 23470	0.500 ± 0.40	1.081 ± 0.41	1.035 ± 0.84	1.218 ± 0.33	1.154 ± 0.31
Gm23119	Predicted gene 23119	0.520 ± 0.96	0.803 ± 0.09	1.011 ± 0.23	1.122 ± 0.32	0.974 ± 0.32
Prr11	Proline rich 11	0.521 ± 0.43	1.278 ± 0.33	0.490 ± 0.14	2.254 ± 0.26	2.306 ± 0.26
Tmem47	Transmembrane protein 47	0.542 ± 0.26	1.259 ± 0.27	0.720 ± 0.12	0.721 ± 0.09	0.831 ± 0.16
Gm24655	Predicted gene 24655	0.545 ± 0.40	0.835 ± 0.43	1.605 ± 1.08	0.682 ± 0.53	0.782 ± 0.54
Hist1h3e	Histone cluster 1, H3e	0.547 ± 0.43	0.905 ± 0.29	0.589 ± 0.50	2.213 ± 0.61	2.474 ± 0.57
Gm22303	Predicted gene 22303	0.559 ± 0.29	1.005 ± 0.29	0.581 ± 0.39	0.964 ± 0.35	1.016 ± 0.32
mt-Tk	Mitochondrially encoded tRNA lysine	0.559 ± 0.48	0.757 ± 0.43	0.688 ± 0.65	0.938 ± 0.31	1.047 ± 0.38
Traj54	T cell receptor alpha joining 54	0.559 ± 0.71	0.799 ± 0.28	0.635 ± 0.44	1.164 ± 0.46	1.332 ± 0.53
Emp1	Epithelial membrane protein 1	0.561 ± 0.48	1.444 ± 0.29	0.711 ± 0.16	1.143 ± 0.19	0.990 ± 0.21
Hibadh	3-hydroxyisobutyrate dehydrogenase	0.565 ± 0.77	1.097 ± 0.78	0.504 ± 1.10	0.969 ± 0.70	1.090 ± 0.84
Gm22973	Predicted gene 22973	0.582 ± 0.32	0.922 ± 0.86	1.708 ± 1.30	1.075 ± 0.65	1.189 ± 0.96
Lgals9	Lectin, galactose binding, soluble 9	0.587 ± 0.47	1.079 ± 0.21	1.781 ± 0.29	1.151 ± 0.27	1.264 ± 0.26
Samd5	Sterile alpha motif domain containing 5	0.587 ± 0.17	2.183 ± 0.27	1.122 ± 0.16	1.149 ± 0.32	1.310 ± 0.25
Egr1	Early growth response 1	0.590 ± 0.59	1.384 ± 0.24	1.412 ± 0.31	1.696 ± 0.24	1.882 ± 0.23
Gtse1	G two S phase expressed protein 1	0.593 ± 0.35	1.712 ± 0.27	1.588 ± 0.35	1.908 ± 0.26	1.735 ± 0.24
Gm22358	Predicted gene 22358	0.595 ± 0.20	1.069 ± 0.22	0.656 ± 0.28	1.017 ± 0.26	1.077 ± 0.25
Kifc1	Kinesin family member C1	0.601 ± 0.69	1.211 ± 0.43	0.695 ± 0.22	1.200 ± 0.42	1.249 ± 0.39
Inhba	Inhibin beta-A	0.603 ± 0.41	1.688 ± 0.27	4.050 ± 0.45	2.449 ± 0.44	1.465 ± 0.38
Gm24519	Predicted gene 24519	0.605 ± 0.50	0.658 ± 0.46	1.406 ± 0.29	0.773 ± 0.58	0.874 ± 0.54
Gm24679	Predicted gene 24679	0.607 ± 0.63	0.514 ± 0.56	0.902 ± 0.16	0.949 ± 0.41	1.124 ± 0.38
Tnfrsf10b	Tumor necrosis factor receptor superfamily, member 10b	0.609 ± 0.23	1.342 ± 0.22	1.533 ± 0.16	1.049 ± 0.19	0.915 ± 0.19
E2f8	E2F transcription factor 8	0.611 ± 0.22	1.001 ± 0.12	0.157 ± 0.28	1.127 ± 0.27	1.227 ± 0.29

Hist1h2ai	Histone cluster 1, H2ai	0.612 ± 0.28	1.226 ± 0.19	0.823 ± 0.29	1.397 ± 0.38	1.946 ± 0.43
Glipr1	GLI pathogenesis-related 1	0.612 ± 0.62	2.600 ± 0.56	4.190 ± 0.30	5.148 ± 0.55	3.606 ± 0.54
Tgfb1i1	Transforming growth factor beta 1 induced transcript 1	0.614 ± 0.51	0.729 ± 0.22	1.000 ± 0.19	0.861 ± 0.16	0.837 ± 0.16
Crip2	Cysteine rich protein 2	0.616 ± 0.19	1.464 ± 0.20	1.331 ± 0.12	1.481 ± 0.17	1.403 ± 0.17
Perp	PERP, TP53 apoptosis effector	0.617 ± 0.36	2.334 ± 0.40	1.493 ± 0.23	0.798 ± 0.32	0.667 ± 0.26
Gm2573	Predicted gene 2573	0.618 ± 0.59	0.695 ± 0.14	0.875 ± 0.09	0.816 ± 0.09	0.867 ± 0.17
Olfr331	Olfactory receptor 331	0.620 ± 0.37	0.739 ± 0.40	1.099 ± 0.44	1.047 ± 0.47	1.170 ± 0.50
Hist1h3d	Histone cluster 1, H3d	0.621 ± 0.26	1.500 ± 0.21	0.827 ± 0.53	1.411 ± 0.68	1.916 ± 0.60
Gm23130	Predicted gene 23130	0.622 ± 0.23	0.745 ± 0.15	0.791 ± 0.32	0.985 ± 0.11	1.082 ± 0.11
1700007K13Rik	RIKEN cDNA 1700007K13 gene	0.622 ± 0.18	1.892 ± 0.45	3.677 ± 0.25	0.956 ± 0.34	1.126 ± 0.29
Mir30e	MicroRNA 30e	0.626 ± 0.41	0.596 ± 0.54	1.078 ± 0.44	1.207 ± 0.73	1.103 ± 0.91
Gm23058	Predicted gene 23058	0.629 ± 0.40	0.971 ± 0.37	1.124 ± 0.63	1.021 ± 0.43	1.016 ± 0.40
Gm7665	Predicted pseudogene 7665	0.629 ± 0.63	0.902 ± 0.40	1.265 ± 0.32	1.204 ± 0.41	0.973 ± 0.49
Sfxn3	Sideroflexin 3	0.630 ± 0.34	1.007 ± 0.09	1.932 ± 0.11	1.515 ± 0.16	1.388 ± 0.18
Ddias	DNA damage-induced apoptosis suppressor	0.632 ± 0.58	2.713 ± 0.38	2.248 ± 0.33	2.074 ± 0.44	1.852 ± 0.32
Gm25394	Predicted gene 25394	0.632 ± 0.14	0.556 ± 0.12	0.902 ± 0.22	1.002 ± 1.94	1.010 ± 1.99
Rnu3a	U3A small nuclear RNA	0.634 ± 0.17	0.681 ± 0.14	1.428 ± 0.33	1.627 ± 1.25	1.345 ± 1.28
Nop56	NOP56 ribonucleoprotein	0.635 ± 0.20	0.722 ± 0.21	0.768 ± 0.68	0.954 ± 0.53	0.949 ± 0.53
Fhdc1	FH2 domain containing 1	0.635 ± 0.41	1.791 ± 0.38	0.804 ± 0.13	0.907 ± 0.12	0.968 ± 0.16
Actg1	Actin, gamma, cytoplasmic 1	0.637 ± 0.33	0.864 ± 0.33	0.990 ± 0.53	0.932 ± 0.25	0.902 ± 0.19
Gm13770	Predicted gene 13770	0.638 ± 0.57	0.708 ± 0.09	0.889 ± 0.13	0.827 ± 0.20	0.851 ± 0.22
Cxx1a	CAAX box 1A	0.639 ± 0.49	0.745 ± 0.26	1.051 ± 0.21	1.073 ± 0.38	0.890 ± 0.48
Itga3	Integrin alpha 3	0.639 ± 0.34	1.456 ± 0.28	0.888 ± 0.13	0.810 ± 0.18	0.767 ± 0.17
Slc18b1	Solute carrier family 18, subfamily B, member 1	0.640 ± 0.30	0.705 ± 0.12	1.421 ± 0.19	1.044 ± 0.37	1.094 ± 0.37
Olfr147	Olfactory receptor 147	0.642 ± 0.37	0.718 ± 0.34	0.868 ± 0.41	0.956 ± 0.43	0.843 ± 0.37
Stil	Scl/Tal1 interrupting locus	0.643 ± 0.47	1.420 ± 0.24	0.537 ± 0.21	1.871 ± 0.36	1.884 ± 0.37
Olfr710	Olfactory receptor 710	0.643 ± 0.29	0.627 ± 0.31	1.128 ± 0.19	1.143 ± 0.28	1.186 ± 0.25
Jph2	Junctophilin 2	0.643 ± 0.47	0.802 ± 0.25	1.119 ± 0.28	0.496 ± 0.26	0.677 ± 0.35
Wdr46	WD repeat domain 46	0.644 ± 0.40	0.904 ± 0.10	0.756 ± 0.08	0.858 ± 0.23	0.949 ± 0.10

Ccnb2	Cyclin B2	0.645 ± 0.24	1.125 ± 0.16	0.362 ± 0.19	2.194 ± 0.24	1.969 ± 0.23
Krt17	Keratin 17	0.645 ± 0.99	0.963 ± 0.59	0.513 ± 0.36	0.958 ± 0.62	0.705 ± 0.78
Dsp	Desmoplakin	0.646 ± 0.60	2.182 ± 0.63	0.691 ± 0.26	1.744 ± 0.35	0.778 ± 0.24
Kctd12	Potassium channel tetramerisation domain	0.648 ± 0.60	2.099 ± 0.50	3.137 ± 0.45	1.773 ± 0.23	1.982 ± 0.18
Ly6e	Lymphocyte antigen 6 complex, locus E	0.648 ± 0.28	1.710 ± 0.22	1.676 ± 0.18	2.260 ± 0.40	1.860 ± 0.43
Cd44	CD44 antigen	0.649 ± 0.41	2.530 ± 0.42	2.583 ± 0.13	2.607 ± 0.18	2.334 ± 0.23
Fam198b	Family with sequence similarity 198, member B	0.649 ± 0.55	0.978 ± 0.33	0.746 ± 0.17	0.426 ± 0.21	0.516 ± 0.23
G530011006 Rik	RIKEN cDNA G530011O06 gene	0.649 ± 0.21	0.987 ± 0.19	1.39 ± 0.35	1.353 ± 0.55	1.372 ± 0.23
Gm26443	Predicted gene 26443	0.651 ± 0.50	1.123 ± 0.71	0.929 ± 0.63	0.981 ± 0.48	0.759 ± 0.53
Gm20634	Predicted gene 20634	0.651 ± 0.23	0.766 ± 0.14	1.147 ± 0.19	1.086 ± 0.29	1.049 ± 0.41
Trav13d-4	T cell receptor alpha variable 13D-4	0.651 ± 0.40	0.634 ± 0.63	0.788 ± 0.45	0.95 ± 0.34	1.001 ± 0.32
Kitl	Kit ligand	0.653 ± 0.39	4.790 ± 0.40	5.222 ± 0.44	2.417 ± 0.34	1.719 ± 0.41
Vps33b	Vacuolar protein sorting 33B	0.653 ± 0.30	0.832 ± 0.06	1.011 ± 0.13	1.048 ± 0.29	0.971 ± 0.19
Rela	v-rel reticuloendotheliosis viral oncogene homolog A	0.653 ± 0.28	0.958 ± 0.05	1.381 ± 0.17	1.162 ± 0.15	1.033 ± 0.15
Gm23237	Predicted gene 23237	0.654 ± 0.37	0.746 ± 0.40	0.897 ± 0.33	1.179 ± 1.04	1.37 ± 0.76
Bax	BCL2-associated X protein	0.655 ± 0.58	1.221 ± 0.14	1.633 ± 0.07	1.134 ± 0.14	1.036 ± 0.17
Gm23272	Predicted gene 23272	0.656 ± 0.60	0.969 ± 0.65	0.774 ± 0.38	0.946 ± 0.45	1.104 ± 0.49
Sdpr	Serum deprivation response	0.656 ± 0.43	1.914 ± 0.48	1.905 ± 0.24	0.712 ± 0.37	0.805 ± 0.37
Elovl7	ELOVL family member 7	0.657 ± 0.30	1.610 ± 0.39	1.205 ± 0.35	1.011 ± 0.20	1.027 ± 0.28
Hist1h4d	Histone cluster 1, H4d	0.659 ± 0.82	1.006 ± 0.53	0.624 ± 0.90	1.399 ± 1.01	1.571 ± 1.06
Hist1h4i	Histone cluster 1, H4i	0.660 ± 0.57	1.673 ± 0.30	1.055 ± 0.43	1.715 ± 0.55	1.948 ± 0.48
Ndc80	NDC80 homolog, kinetochore complex component	0.661 ± 0.42	1.326 ± 0.40	0.317 ± 0.29	2.413 ± 0.47	2.693 ± 0.45
Msrb3	Methionine sulfoxide reductase B3	0.662 ± 0.18	0.846 ± 0.18	0.817 ± 0.06	0.899 ± 0.16	0.835 ± 0.16
Id1	Inhibitor of DNA binding 1	0.663 ± 0.27	1.091 ± 0.16	0.973 ± 0.16	0.616 ± 0.19	0.914 ± 0.27
Sfrp1	Secreted frizzled-related protein 1	0.663 ± 0.22	1.042 ± 0.14	0.814 ± 0.29	0.909 ± 0.07	1.031 ± 0.07
Pqlc3	PQ loop repeat containing	0.664 ± 0.28	1.674 ± 0.27	2.827 ± 0.27	1.483 ± 0.26	1.135 ± 0.27
B4galt1	UDP-Gal:betaGlcNAc beta 1,4-galactosyltransferase	0.664 ± 0.08	1.009 ± 0.07	1.894 ± 0.23	2.169 ± 0.16	1.788 ± 0.17
Mcm5	Minichromosome maintenance deficient 5, cell division cycle 4	0.665 ± 0.47	1.462 ± 0.19	0.459 ± 0.18	1.763 ± 0.25	1.772 ± 0.21
Dusp1	Dual specificity phosphatase 1	0.666 ± 0.42	0.977 ± 0.28	1.841 ± 0.31	0.989 ± 0.23	1.034 ± 0.31

Gm22672	Predicted gene 22672	0.667 ± 0.46	0.934 ± 0.29	1.269 ± 0.16	1.157 ± 0.46	1.023 ± 0.47
Gm12185	Predicted gene 12185	0.668 ± 0.38	0.924 ± 0.29	1.017 ± 0.39	0.981 ± 0.39	1.076 ± 0.40
Angptl7	Angiopoietin-like 7	0.668 ± 0.5	0.714 ± 0.36	1.333 ± 0.35	0.804 ± 0.37	0.951 ± 0.41
Arl6ip1	ADP-ribosylation factor-like 6 interacting protein 1	0.669 ± 0.19	1.292 ± 0.08	0.965 ± 0.12	1.451 ± 0.12	1.428 ± 0.14
Gm25188	Predicted gene 25188	0.669 ± 0.30	1.001 ± 0.27	0.814 ± 0.50	1.038 ± 0.25	1.012 ± 0.26
Zfp365	Zinc finger protein 365	0.669 ± 0.20	1.172 ± 0.13	1.422 ± 0.20	0.803 ± 0.16	0.836 ± 0.15
Krtap19-9b	Keratin associated protein 19-9B	0.670 ± 0.24	0.665 ± 0.30	1.074 ± 0.41	1.029 ± 0.40	1.340 ± 0.54
Nes	Nestin	0.671 ± 0.59	1.457 ± 0.48	0.493 ± 0.24	0.773 ± 0.41	0.861 ± 0.36
9030617O03 Rik	RIKEN cDNA 9030617O03 gene	0.672 ± 0.41	1.823 ± 0.26	3.105 ± 0.32	1.376 ± 0.28	1.071 ± 0.27
Gm23406	Predicted gene 23406	0.672 ± 0.32	1.237 ± 0.37	0.764 ± 0.49	0.917 ± 0.54	0.924 ± 0.63
Gm25776	Predicted gene 25776	0.673 ± 0.32	0.811 ± 0.29	0.784 ± 0.85	1.072 ± 1.69	0.937 ± 1.51
Sncg	Synuclein, gamma	0.673 ± 0.49	2.779 ± 0.55	1.759 ± 0.33	1.876 ± 0.59	1.653 ± 0.47
Gm23513	Predicted gene 23513	0.674 ± 0.28	0.719 ± 0.19	1.047 ± 0.25	0.984 ± 0.78	1.005 ± 0.67
Gm15726	Predicted gene 15726	0.675 ± 0.26	1.302 ± 0.47	0.985 ± 0.08	1.084 ± 0.69	1.180 ± 0.48
Rab39b	RAB39B, member RAS oncogene family	0.675 ± 0.17	0.630 ± 0.15	0.327 ± 0.16	0.543 ± 0.21	0.625 ± 0.33
Dusp6	Dual specificity phosphatase 6	0.675 ± 0.48	3.195 ± 0.37	2.666 ± 0.22	2.388 ± 0.52	2.428 ± 0.49
Abcb1b	ATP-binding cassette, sub-family B member 1B	0.675 ± 0.58	2.438 ± 0.54	1.792 ± 0.44	1.478 ± 0.37	1.339 ± 0.26
Twist2	Twist basic helix-loop-helix transcription factor 2	0.675 ± 0.36	1.298 ± 0.20	0.905 ± 0.39	1.276 ± 0.47	1.361 ± 0.48
Gm25970	Predicted gene 25970	0.675 ± 0.37	0.760 ± 0.35	1.136 ± 0.92	1.190 ± 1.35	1.198 ± 1.31
Unc13c	Unc-13 homolog	0.675 ± 0.42	1.794 ± 0.60	0.846 ± 0.28	0.882 ± 0.30	1.010 ± 0.14
Olfr870	Olfactory receptor 870	0.675 ± 0.42	0.676 ± 0.22	0.984 ± 0.26	0.904 ± 0.29	1.031 ± 0.18
Lrrn3	Leucine rich repeat protein 3, neuronal	0.675 ± 0.51	3.068 ± 0.61	0.979 ± 0.30	1.700 ± 0.30	2.129 ± 0.25
Lrrc61	Leucine rich repeat containing 61	0.676 ± 0.25	0.537 ± 0.21	0.708 ± 0.14	0.747 ± 0.18	0.880 ± 0.15
Hist1h4f	Histone cluster 1, H4f	0.677 ± 0.22	0.855 ± 0.23	0.955 ± 0.38	0.934 ± 0.29	0.983 ± 0.39
9230114K14 Rik	RIKEN cDNA 9230114K14 gene	0.677 ± 0.24	1.402 ± 0.31	3.265 ± 0.39	1.316 ± 0.23	1.169 ± 0.23
Fam83h	Family with sequence similarity 83, member H	0.678 ± 0.43	1.077 ± 0.26	1.096 ± 0.12	0.966 ± 0.13	0.891 ± 0.27
H2afx	H2A histone family, member X	0.678 ± 0.37	1.294 ± 0.08	0.397 ± 0.24	1.639 ± 0.11	1.597 ± 0.13
Mir344e	MicroRNA 344e	0.679 ± 0.49	0.697 ± 0.51	0.863 ± 0.43	0.916 ± 0.58	0.987 ± 0.57
Abi2	Abl-interactor 2	0.680 ± 0.04	0.910 ± 0.06	0.979 ± 0.14	0.760 ± 0.11	0.838 ± 0.04

Masp1	Mannan-binding lectin serine peptidase 1	0.680 ± 0.49	2.061 ± 0.50	1.639 ± 0.26	1.277 ± 0.32	1.252 ± 0.36
Gm25762	Predicted gene 25762	0.681 ± 0.52	0.877 ± 0.47	0.732 ± 0.59	1.026 ± 0.32	1.003 ± 0.52
Gm25443	Predicted gene 25443	0.681 ± 0.69	0.894 ± 0.18	0.977 ± 0.10	1.058 ± 0.27	0.989 ± 0.21
Krtap1-5	Keratin associated protein 1-5	0.681 ± 0.43	1.127 ± 0.17	0.770 ± 0.29	0.987 ± 0.44	1.232 ± 0.58
Pparg	Peroxisome proliferator activated receptor gamma	0.681 ± 0.43	1.899 ± 0.46	1.029 ± 0.21	0.656 ± 0.22	0.827 ± 0.23
Osr1	Odd-skipped related 1	0.682 ± 0.14	0.701 ± 0.18	1.892 ± 0.17	0.894 ± 0.44	1.021 ± 0.49
Taf9b	TAF9B RNA polymerase II, TBP-associated factor	0.682 ± 0.27	0.666 ± 0.28	0.490 ± 0.20	0.731 ± 0.14	0.622 ± 0.16
P4ha3	Procollagen-proline, 2-oxoglutarate 4-dioxygenase	0.682 ± 0.23	0.629 ± 0.15	0.317 ± 0.20	0.375 ± 0.21	0.500 ± 0.22
Hist1h4n	Histone cluster 1, H4n	0.683 ± 0.44	1.412 ± 0.48	1.115 ± 0.62	1.940 ± 0.66	1.891 ± 0.40
Creb3l1	cAMP responsive element binding protein 3-like 1	0.683 ± 0.25	0.564 ± 0.26	1.315 ± 0.04	1.150 ± 0.13	1.090 ± 0.13
P3h2	Prolyl 3-hydroxylase 2	0.685 ± 0.04	0.908 ± 0.10	0.490 ± 0.18	0.778 ± 0.28	0.600 ± 0.17
Lce3c	Late cornified envelope 3C	0.686 ± 0.82	1.097 ± 0.62	1.578 ± 0.82	1.272 ± 0.34	1.575 ± 0.22
Plk1	Polo-like kinase 1	0.686 ± 0.30	1.284 ± 0.20	0.355 ± 0.14	2.139 ± 0.12	2.048 ± 0.13
Tagln	Transgelin	0.687 ± 0.70	0.660 ± 0.38	0.641 ± 0.50	0.412 ± 0.21	0.545 ± 0.23
Jun	Jun proto-oncogene	0.687 ± 0.39	1.689 ± 0.19	2.359 ± 0.16	1.492 ± 0.31	1.090 ± 0.21
Gm22327	Predicted gene 22327	0.687 ± 0.49	0.920 ± 0.39	1.107 ± 0.85	1.410 ± 0.42	1.625 ± 0.47
Ctxn1	Cortexin 1	0.687 ± 0.06	1.342 ± 0.09	2.261 ± 0.26	1.324 ± 0.27	1.390 ± 0.21
LOC105244345	Small nuclear ribonucleoprotein Sm D1-like	0.688 ± 0.34	0.947 ± 0.26	0.677 ± 0.39	0.869 ± 0.20	0.905 ± 0.21
Eif2s3x	Eukaryotic translation initiation factor 2, subunit 3	0.688 ± 0.29	0.999 ± 0.16	0.937 ± 0.17	0.888 ± 0.26	0.902 ± 0.27
Zwilch	Zwilch kinetochore protein	0.688 ± 0.37	1.093 ± 0.17	0.434 ± 0.12	1.471 ± 0.21	1.499 ± 0.24
Wdr83os	WD repeat domain 83 opposite strand	0.689 ± 0.16	0.909 ± 0.08	0.920 ± 0.31	0.974 ± 0.27	1.143 ± 0.24
Ifit1bl2	Interferon induced protein with tetratricopeptide repeats 1B	0.689 ± 0.28	0.767 ± 0.2	0.773 ± 0.2	0.896 ± 0.32	0.937 ± 0.32
Ska3	Spindle and kinetochore associated complex subunit 3	0.689 ± 0.51	1.749 ± 0.32	0.431 ± 0.29	1.896 ± 0.42	1.978 ± 0.36
Ifi44	Interferon-induced protein 44	0.690 ± 0.19	1.986 ± 1.28	0.538 ± 0.18	0.463 ± 0.24	0.640 ± 0.26
Fxyd5	FXYD domain-containing ion transport regulator 5	0.691 ± 0.15	1.496 ± 0.19	1.455 ± 0.19	2.509 ± 0.38	1.541 ± 0.33
Gm11487	Predicted gene 11487	0.691 ± 0.56	0.690 ± 0.54	1.091 ± 0.59	1.300 ± 0.50	1.138 ± 0.40
Ibsp	Integrin binding sialoprotein	0.692 ± 0.26	0.739 ± 0.26	2.754 ± 0.84	1.205 ± 0.18	1.493 ± 0.25
Bub1	Budding uninhibited by benzimidazoles 1 homolog	0.692 ± 0.52	1.44 ± 0.29	0.369 ± 0.22	2.425 ± 0.3	2.342 ± 0.29
D430020J02 Rik	RIKEN cDNA D430020J02 gene	0.693 ± 0.30	1.046 ± 0.11	0.653 ± 0.12	1.250 ± 0.09	1.125 ± 0.14

Gcnt4	Glucosaminyl transferase 4, core 2	0.694 ± 0.36	2.182 ± 0.35	1.202 ± 0.44	1.781 ± 0.49	1.501 ± 0.32
Gm17019	Predicted gene 17019	0.694 ± 0.17	0.601 ± 0.30	1.058 ± 0.40	1.167 ± 0.48	1.416 ± 0.59
Fam111a	Family with sequence similarity 111, member A	0.695 ± 0.49	2.158 ± 0.38	0.688 ± 0.21	3.163 ± 0.35	3.350 ± 0.36
Rhou	Ras homolog gene family, member U	0.695 ± 0.32	1.136 ± 0.23	1.600 ± 0.22	0.993 ± 0.22	1.217 ± 0.31
Shcbp1	Shc SH2-domain binding protein 1	0.695 ± 0.62	1.338 ± 0.44	0.310 ± 0.12	2.813 ± 0.20	2.768 ± 0.28
Cbll1	Casitas B-lineage lymphoma-like 1	0.695 ± 0.19	1.045 ± 0.20	1.072 ± 0.16	0.948 ± 0.13	0.970 ± 0.09
Lsm2	LSM2 homolog, U6 small nuclear RNA associated	0.695 ± 0.40	1.247 ± 0.19	0.702 ± 0.31	1.038 ± 0.25	1.037 ± 0.15
Tmem74	Transmembrane protein 74	0.695 ± 0.13	2.854 ± 0.21	3.009 ± 0.29	2.863 ± 0.56	1.537 ± 0.40
Cdc6	Cell division cycle 6	0.696 ± 0.45	1.241 ± 0.23	0.637 ± 0.16	1.737 ± 0.32	1.928 ± 0.17
Art2b	ADP-ribosyltransferase 2b	0.697 ± 0.31	0.752 ± 0.23	1.140 ± 0.25	0.993 ± 0.52	1.054 ± 0.38
Arhgap19	Rho GTPase activating protein 19	0.698 ± 0.38	1.160 ± 0.27	0.690 ± 0.22	1.435 ± 0.21	1.424 ± 0.19
Sorbs3	Sorbin and SH3 domain containing 3	0.699 ± 0.29	0.751 ± 0.13	0.915 ± 0.07	1.002 ± 0.21	0.925 ± 0.24
Hist1h2bk	Histone cluster 1, H2bk	0.699 ± 0.44	1.123 ± 0.14	0.272 ± 0.53	2.002 ± 0.41	2.190 ± 0.57
Gchfr	GTP cyclohydrolase I feedback regulator	0.700 ± 0.16	1.447 ± 0.33	1.214 ± 0.30	1.314 ± 0.24	1.247 ± 0.25
